# Search for dark matter in events with heavy quarks and missing transverse momentum in $pp$ collisions with the ATLAS detector

**DOI:** 10.1140/epjc/s10052-015-3306-z

**Published:** 2015-02-24

**Authors:** ATLAS Collaboration, G. Aad, B. Abbott, J. Abdallah, S. Abdel Khalek, O. Abdinov, R. Aben, B. Abi, M. Abolins, O. S. AbouZeid, H. Abramowicz, H. Abreu, R. Abreu, Y. Abulaiti, B. S. Acharya, L. Adamczyk, D. L. Adams, J. Adelman, S. Adomeit, T. Adye, T. Agatonovic-Jovin, J. A. Aguilar-Saavedra, M. Agustoni, S. P. Ahlen, F. Ahmadov, G. Aielli, H. Akerstedt, T. P. A. Åkesson, G. Akimoto, A. V. Akimov, G. L. Alberghi, J. Albert, S. Albrand, M. J. Alconada Verzini, M. Aleksa, I. N. Aleksandrov, C. Alexa, G. Alexander, G. Alexandre, T. Alexopoulos, M. Alhroob, G. Alimonti, L. Alio, J. Alison, B. M. M. Allbrooke, L. J. Allison, P. P. Allport, A. Aloisio, A. Alonso, F. Alonso, C. Alpigiani, A. Altheimer, B. Alvarez Gonzalez, M. G. Alviggi, K. Amako, Y. Amaral Coutinho, C. Amelung, D. Amidei, S. P. Amor Dos Santos, A. Amorim, S. Amoroso, N. Amram, G. Amundsen, C. Anastopoulos, L. S. Ancu, N. Andari, T. Andeen, C. F. Anders, G. Anders, K. J. Anderson, A. Andreazza, V. Andrei, X. S. Anduaga, S. Angelidakis, I. Angelozzi, P. Anger, A. Angerami, F. Anghinolfi, A. V. Anisenkov, N. Anjos, A. Annovi, M. Antonaki, M. Antonelli, A. Antonov, J. Antos, F. Anulli, M. Aoki, L. Aperio Bella, R. Apolle, G. Arabidze, I. Aracena, Y. Arai, J. P. Araque, A. T. H. Arce, F. A. Arduh, J-F. Arguin, S. Argyropoulos, M. Arik, A. J. Armbruster, O. Arnaez, V. Arnal, H. Arnold, M. Arratia, O. Arslan, A. Artamonov, G. Artoni, S. Asai, N. Asbah, A. Ashkenazi, B. Åsman, L. Asquith, K. Assamagan, R. Astalos, M. Atkinson, N. B. Atlay, B. Auerbach, K. Augsten, M. Aurousseau, G. Avolio, B. Axen, G. Azuelos, Y. Azuma, M. A. Baak, A. E. Baas, C. Bacci, H. Bachacou, K. Bachas, M. Backes, M. Backhaus, E. Badescu, P. Bagiacchi, P. Bagnaia, Y. Bai, T. Bain, J. T. Baines, O. K. Baker, P. Balek, F. Balli, E. Banas, Sw. Banerjee, A. A. E. Bannoura, H. S. Bansil, L. Barak, S. P. Baranov, E. L. Barberio, D. Barberis, M. Barbero, T. Barillari, M. Barisonzi, T. Barklow, N. Barlow, S. L. Barnes, B. M. Barnett, R. M. Barnett, Z. Barnovska, A. Baroncelli, G. Barone, A. J. Barr, F. Barreiro, J. Barreiro Guimarães da Costa, R. Bartoldus, A. E. Barton, P. Bartos, V. Bartsch, A. Bassalat, A. Basye, R. L. Bates, S. J. Batista, J. R. Batley, M. Battaglia, M. Battistin, F. Bauer, H. S. Bawa, J. B. Beacham, M. D. Beattie, T. Beau, P. H. Beauchemin, R. Beccherle, P. Bechtle, H. P. Beck, K. Becker, S. Becker, M. Beckingham, C. Becot, A. J. Beddall, A. Beddall, S. Bedikian, V. A. Bednyakov, C. P. Bee, L. J. Beemster, T. A. Beermann, M. Begel, K. Behr, C. Belanger-Champagne, P. J. Bell, W. H. Bell, G. Bella, L. Bellagamba, A. Bellerive, M. Bellomo, K. Belotskiy, O. Beltramello, O. Benary, D. Benchekroun, K. Bendtz, N. Benekos, Y. Benhammou, E. Benhar Noccioli, J. A. Benitez Garcia, D. P. Benjamin, J. R. Bensinger, S. Bentvelsen, D. Berge, E. Bergeaas Kuutmann, N. Berger, F. Berghaus, J. Beringer, C. Bernard, P. Bernat, C. Bernius, F. U. Bernlochner, T. Berry, P. Berta, C. Bertella, G. Bertoli, F. Bertolucci, C. Bertsche, D. Bertsche, M. I. Besana, G. J. Besjes, O. Bessidskaia Bylund, M. Bessner, N. Besson, C. Betancourt, S. Bethke, W. Bhimji, R. M. Bianchi, L. Bianchini, M. Bianco, O. Biebel, S. P. Bieniek, K. Bierwagen, J. Biesiada, M. Biglietti, J. Bilbao De Mendizabal, H. Bilokon, M. Bindi, S. Binet, A. Bingul, C. Bini, C. W. Black, J. E. Black, K. M. Black, D. Blackburn, R. E. Blair, J.-B. Blanchard, T. Blazek, I. Bloch, C. Blocker, W. Blum, U. Blumenschein, G. J. Bobbink, V. S. Bobrovnikov, S. S. Bocchetta, A. Bocci, C. Bock, C. R. Boddy, M. Boehler, T. T. Boek, J. A. Bogaerts, A. G. Bogdanchikov, A. Bogouch, C. Bohm, V. Boisvert, T. Bold, V. Boldea, A. S. Boldyrev, M. Bomben, M. Bona, M. Boonekamp, A. Borisov, G. Borissov, M. Borri, S. Borroni, J. Bortfeldt, V. Bortolotto, K. Bos, D. Boscherini, M. Bosman, H. Boterenbrood, J. Boudreau, J. Bouffard, E. V. Bouhova-Thacker, D. Boumediene, C. Bourdarios, N. Bousson, S. Boutouil, A. Boveia, J. Boyd, I. R. Boyko, I. Bozic, J. Bracinik, A. Brandt, G. Brandt, O. Brandt, U. Bratzler, B. Brau, J. E. Brau, H. M. Braun, S. F. Brazzale, B. Brelier, K. Brendlinger, A. J. Brennan, R. Brenner, S. Bressler, K. Bristow, T. M. Bristow, D. Britton, F. M. Brochu, I. Brock, R. Brock, J. Bronner, G. Brooijmans, T. Brooks, W. K. Brooks, J. Brosamer, E. Brost, J. Brown, P. A. Bruckman de Renstrom, D. Bruncko, R. Bruneliere, S. Brunet, A. Bruni, G. Bruni, M. Bruschi, L. Bryngemark, T. Buanes, Q. Buat, F. Bucci, P. Buchholz, A. G. Buckley, S. I. Buda, I. A. Budagov, F. Buehrer, L. Bugge, M. K. Bugge, O. Bulekov, A. C. Bundock, H. Burckhart, S. Burdin, B. Burghgrave, S. Burke, I. Burmeister, E. Busato, D. Büscher, V. Büscher, P. Bussey, C. P. Buszello, B. Butler, J. M. Butler, A. I. Butt, C. M. Buttar, J. M. Butterworth, P. Butti, W. Buttinger, A. Buzatu, M. Byszewski, S. Cabrera Urbán, D. Caforio, O. Cakir, P. Calafiura, A. Calandri, G. Calderini, P. Calfayan, L. P. Caloba, D. Calvet, S. Calvet, R. Camacho Toro, S. Camarda, D. Cameron, L. M. Caminada, R. Caminal Armadans, S. Campana, M. Campanelli, A. Campoverde, V. Canale, A. Canepa, M. Cano Bret, J. Cantero, R. Cantrill, T. Cao, M. D. M. Capeans Garrido, I. Caprini, M. Caprini, M. Capua, R. Caputo, R. Cardarelli, T. Carli, G. Carlino, L. Carminati, S. Caron, E. Carquin, G. D. Carrillo-Montoya, J. R. Carter, J. Carvalho, D. Casadei, M. P. Casado, M. Casolino, E. Castaneda-Miranda, A. Castelli, V. Castillo Gimenez, N. F. Castro, P. Catastini, A. Catinaccio, J. R. Catmore, A. Cattai, G. Cattani, J. Caudron, V. Cavaliere, D. Cavalli, M. Cavalli-Sforza, V. Cavasinni, F. Ceradini, B. C. Cerio, K. Cerny, A. S. Cerqueira, A. Cerri, L. Cerrito, F. Cerutti, M. Cerv, A. Cervelli, S. A. Cetin, A. Chafaq, D. Chakraborty, I. Chalupkova, P. Chang, B. Chapleau, J. D. Chapman, D. Charfeddine, D. G. Charlton, C. C. Chau, C. A. Chavez Barajas, S. Cheatham, A. Chegwidden, S. Chekanov, S. V. Chekulaev, G. A. Chelkov, M. A. Chelstowska, C. Chen, H. Chen, K. Chen, L. Chen, S. Chen, X. Chen, Y. Chen, H. C. Cheng, Y. Cheng, A. Cheplakov, E. Cheremushkina, R. Cherkaoui El Moursli, V. Chernyatin, E. Cheu, L. Chevalier, V. Chiarella, G. Chiefari, J. T. Childers, A. Chilingarov, G. Chiodini, A. S. Chisholm, R. T. Chislett, A. Chitan, M. V. Chizhov, S. Chouridou, B. K. B. Chow, D. Chromek-Burckhart, M. L. Chu, J. Chudoba, J. J. Chwastowski, L. Chytka, G. Ciapetti, A. K. Ciftci, R. Ciftci, D. Cinca, V. Cindro, A. Ciocio, Z. H. Citron, M. Citterio, M. Ciubancan, A. Clark, P. J. Clark, R. N. Clarke, W. Cleland, J. C. Clemens, C. Clement, Y. Coadou, M. Cobal, A. Coccaro, J. Cochran, L. Coffey, J. G. Cogan, B. Cole, S. Cole, A. P. Colijn, J. Collot, T. Colombo, G. Compostella, P. Conde Muiño, E. Coniavitis, S. H. Connell, I. A. Connelly, S. M. Consonni, V. Consorti, S. Constantinescu, C. Conta, G. Conti, F. Conventi, M. Cooke, B. D. Cooper, A. M. Cooper-Sarkar, N. J. Cooper-Smith, K. Copic, T. Cornelissen, M. Corradi, F. Corriveau, A. Corso-Radu, A. Cortes-Gonzalez, G. Cortiana, G. Costa, M. J. Costa, D. Costanzo, D. Côté, G. Cottin, G. Cowan, B. E. Cox, K. Cranmer, G. Cree, S. Crépé-Renaudin, F. Crescioli, W. A. Cribbs, M. Crispin Ortuzar, M. Cristinziani, V. Croft, G. Crosetti, T. Cuhadar Donszelmann, J. Cummings, M. Curatolo, C. Cuthbert, H. Czirr, P. Czodrowski, S. D’Auria, M. D’Onofrio, M. J. Da Cunha Sargedas De Sousa, C. Da Via, W. Dabrowski, A. Dafinca, T. Dai, O. Dale, F. Dallaire, C. Dallapiccola, M. Dam, A. C. Daniells, M. Danninger, M. Dano Hoffmann, V. Dao, G. Darbo, S. Darmora, J. Dassoulas, A. Dattagupta, W. Davey, C. David, T. Davidek, E. Davies, M. Davies, O. Davignon, A. R. Davison, P. Davison, Y. Davygora, E. Dawe, I. Dawson, R. K. Daya-Ishmukhametova, K. De, R. de Asmundis, S. De Castro, S. De Cecco, N. De Groot, P. de Jong, H. De la Torre, F. De Lorenzi, L. De Nooij, D. De Pedis, A. De Salvo, U. De Sanctis, A. De Santo, J. B. De Vivie De Regie, W. J. Dearnaley, R. Debbe, C. Debenedetti, B. Dechenaux, D. V. Dedovich, I. Deigaard, J. Del Peso, T. Del Prete, F. Deliot, C. M. Delitzsch, M. Deliyergiyev, A. Dell’Acqua, L. Dell’Asta, M. Dell’Orso, M. Della Pietra, D. della Volpe, M. Delmastro, P. A. Delsart, C. Deluca, D. A. DeMarco, S. Demers, M. Demichev, A. Demilly, S. P. Denisov, D. Derendarz, J. E. Derkaoui, F. Derue, P. Dervan, K. Desch, C. Deterre, P. O. Deviveiros, A. Dewhurst, S. Dhaliwal, A. Di Ciaccio, L. Di Ciaccio, A. Di Domenico, C. Di Donato, A. Di Girolamo, B. Di Girolamo, A. Di Mattia, B. Di Micco, R. Di Nardo, A. Di Simone, R. Di Sipio, D. Di Valentino, F. A. Dias, M. A. Diaz, E. B. Diehl, J. Dietrich, T. A. Dietzsch, S. Diglio, A. Dimitrievska, J. Dingfelder, P. Dita, S. Dita, F. Dittus, F. Djama, T. Djobava, J. I. Djuvsland, M. A. B. do Vale, D. Dobos, C. Doglioni, T. Doherty, T. Dohmae, J. Dolejsi, Z. Dolezal, B. A. Dolgoshein, M. Donadelli, S. Donati, P. Dondero, J. Donini, J. Dopke, A. Doria, M. T. Dova, A. T. Doyle, M. Dris, J. Dubbert, S. Dube, E. Dubreuil, E. Duchovni, G. Duckeck, O. A. Ducu, D. Duda, A. Dudarev, F. Dudziak, L. Duflot, L. Duguid, M. Dührssen, M. Dunford, H. Duran Yildiz, M. Düren, A. Durglishvili, D. Duschinger, M. Dwuznik, M. Dyndal, J. Ebke, W. Edson, N. C. Edwards, W. Ehrenfeld, T. Eifert, G. Eigen, K. Einsweiler, T. Ekelof, M. El Kacimi, M. Ellert, S. Elles, F. Ellinghaus, N. Ellis, J. Elmsheuser, M. Elsing, D. Emeliyanov, Y. Enari, O. C. Endner, M. Endo, R. Engelmann, J. Erdmann, A. Ereditato, D. Eriksson, G. Ernis, J. Ernst, M. Ernst, J. Ernwein, D. Errede, S. Errede, E. Ertel, M. Escalier, H. Esch, C. Escobar, B. Esposito, A. I. Etienvre, E. Etzion, H. Evans, A. Ezhilov, L. Fabbri, G. Facini, R. M. Fakhrutdinov, S. Falciano, R. J. Falla, J. Faltova, Y. Fang, M. Fanti, A. Farbin, A. Farilla, T. Farooque, S. Farrell, S. M. Farrington, P. Farthouat, F. Fassi, P. Fassnacht, D. Fassouliotis, A. Favareto, L. Fayard, P. Federic, O. L. Fedin, W. Fedorko, S. Feigl, L. Feligioni, C. Feng, E. J. Feng, H. Feng, A. B. Fenyuk, S. Fernandez Perez, S. Ferrag, J. Ferrando, A. Ferrari, P. Ferrari, R. Ferrari, D. E. Ferreira de Lima, A. Ferrer, D. Ferrere, C. Ferretti, A. Ferretto Parodi, M. Fiascaris, F. Fiedler, A. Filipčič, M. Filipuzzi, F. Filthaut, M. Fincke-Keeler, K. D. Finelli, M. C. N. Fiolhais, L. Fiorini, A. Firan, A. Fischer, J. Fischer, W. C. Fisher, E. A. Fitzgerald, M. Flechl, I. Fleck, P. Fleischmann, S. Fleischmann, G. T. Fletcher, G. Fletcher, T. Flick, A. Floderus, L. R. Flores Castillo, M. J. Flowerdew, A. Formica, A. Forti, D. Fortin, D. Fournier, H. Fox, S. Fracchia, P. Francavilla, M. Franchini, S. Franchino, D. Francis, L. Franconi, M. Franklin, M. Fraternali, S. T. French, C. Friedrich, F. Friedrich, D. Froidevaux, J. A. Frost, C. Fukunaga, E. Fullana Torregrosa, B. G. Fulsom, J. Fuster, C. Gabaldon, O. Gabizon, A. Gabrielli, A. Gabrielli, S. Gadatsch, S. Gadomski, G. Gagliardi, P. Gagnon, C. Galea, B. Galhardo, E. J. Gallas, B. J. Gallop, P. Gallus, G. Galster, K. K. Gan, J. Gao, Y. S. Gao, F. M. Garay Walls, F. Garberson, C. García, J. E. García Navarro, M. Garcia-Sciveres, R. W. Gardner, N. Garelli, V. Garonne, C. Gatti, G. Gaudio, B. Gaur, L. Gauthier, P. Gauzzi, I. L. Gavrilenko, C. Gay, G. Gaycken, E. N. Gazis, P. Ge, Z. Gecse, C. N. P. Gee, D. A. A. Geerts, Ch. Geich-Gimbel, K. Gellerstedt, C. Gemme, A. Gemmell, M. H. Genest, S. Gentile, M. George, S. George, D. Gerbaudo, A. Gershon, H. Ghazlane, N. Ghodbane, B. Giacobbe, S. Giagu, V. Giangiobbe, P. Giannetti, F. Gianotti, B. Gibbard, S. M. Gibson, M. Gilchriese, T. P. S. Gillam, D. Gillberg, G. Gilles, D. M. Gingrich, N. Giokaris, M. P. Giordani, R. Giordano, F. M. Giorgi, F. M. Giorgi, P. F. Giraud, D. Giugni, C. Giuliani, M. Giulini, B. K. Gjelsten, S. Gkaitatzis, I. Gkialas, E. L. Gkougkousis, L. K. Gladilin, C. Glasman, J. Glatzer, P. C. F. Glaysher, A. Glazov, G. L. Glonti, M. Goblirsch-Kolb, J. R. Goddard, J. Godlewski, C. Goeringer, S. Goldfarb, T. Golling, D. Golubkov, A. Gomes, L. S. Gomez Fajardo, R. Gonçalo, J. Goncalves Pinto Firmino Da Costa, L. Gonella, S. González de la Hoz, G. Gonzalez Parra, S. Gonzalez-Sevilla, L. Goossens, P. A. Gorbounov, H. A. Gordon, I. Gorelov, B. Gorini, E. Gorini, A. Gorišek, E. Gornicki, A. T. Goshaw, C. Gössling, M. I. Gostkin, M. Gouighri, D. Goujdami, M. P. Goulette, A. G. Goussiou, C. Goy, E. Gozani, H. M. X. Grabas, L. Graber, I. Grabowska-Bold, P. Grafström, K-J. Grahn, J. Gramling, E. Gramstad, S. Grancagnolo, V. Grassi, V. Gratchev, H. M. Gray, E. Graziani, O. G. Grebenyuk, Z. D. Greenwood, K. Gregersen, I. M. Gregor, P. Grenier, J. Griffiths, A. A. Grillo, K. Grimm, S. Grinstein, Ph. Gris, Y. V. Grishkevich, J.-F. Grivaz, J. P. Grohs, A. Grohsjean, E. Gross, J. Grosse-Knetter, G. C. Grossi, Z. J. Grout, L. Guan, J. Guenther, F. Guescini, D. Guest, O. Gueta, C. Guicheney, E. Guido, T. Guillemin, S. Guindon, U. Gul, C. Gumpert, J. Guo, S. Gupta, P. Gutierrez, N. G. Gutierrez Ortiz, C. Gutschow, N. Guttman, C. Guyot, C. Gwenlan, C. B. Gwilliam, A. Haas, C. Haber, H. K. Hadavand, N. Haddad, P. Haefner, S. Hageböeck, Z. Hajduk, H. Hakobyan, M. Haleem, D. Hall, G. Halladjian, G. D. Hallewell, K. Hamacher, P. Hamal, K. Hamano, M. Hamer, A. Hamilton, S. Hamilton, G. N. Hamity, P. G. Hamnett, L. Han, K. Hanagaki, K. Hanawa, M. Hance, P. Hanke, R. Hanna, J. B. Hansen, J. D. Hansen, P. H. Hansen, K. Hara, A. S. Hard, T. Harenberg, F. Hariri, S. Harkusha, D. Harper, R. D. Harrington, O. M. Harris, P. F. Harrison, F. Hartjes, M. Hasegawa, S. Hasegawa, Y. Hasegawa, A. Hasib, S. Hassani, S. Haug, M. Hauschild, R. Hauser, M. Havranek, C. M. Hawkes, R. J. Hawkings, A. D. Hawkins, T. Hayashi, D. Hayden, C. P. Hays, J. M. Hays, H. S. Hayward, S. J. Haywood, S. J. Head, T. Heck, V. Hedberg, L. Heelan, S. Heim, T. Heim, B. Heinemann, L. Heinrich, J. Hejbal, L. Helary, C. Heller, M. Heller, S. Hellman, D. Hellmich, C. Helsens, J. Henderson, Y. Heng, R. C. W. Henderson, C. Hengler, A. Henrichs, A. M. Henriques Correia, S. Henrot-Versille, G. H. Herbert, Y. Hernández Jiménez, R. Herrberg-Schubert, G. Herten, R. Hertenberger, L. Hervas, G. G. Hesketh, N. P. Hessey, R. Hickling, E. Higón-Rodriguez, E. Hill, J. C. Hill, K. H. Hiller, S. J. Hillier, I. Hinchliffe, E. Hines, M. Hirose, D. Hirschbuehl, J. Hobbs, N. Hod, M. C. Hodgkinson, P. Hodgson, A. Hoecker, M. R. Hoeferkamp, F. Hoenig, D. Hoffmann, M. Hohlfeld, T. R. Holmes, T. M. Hong, L. Hooft van Huysduynen, W. H. Hopkins, Y. Horii, A. J. Horton, J-Y. Hostachy, S. Hou, A. Hoummada, J. Howard, J. Howarth, M. Hrabovsky, I. Hristova, J. Hrivnac, T. Hryn’ova, A. Hrynevich, C. Hsu, P. J. Hsu, S.-C. Hsu, D. Hu, X. Hu, Y. Huang, Z. Hubacek, F. Hubaut, F. Huegging, T. B. Huffman, E. W. Hughes, G. Hughes, M. Huhtinen, T. A. Hülsing, M. Hurwitz, N. Huseynov, J. Huston, J. Huth, G. Iacobucci, G. Iakovidis, I. Ibragimov, L. Iconomidou-Fayard, E. Ideal, Z. Idrissi, P. Iengo, O. Igonkina, T. Iizawa, Y. Ikegami, K. Ikematsu, M. Ikeno, Y. Ilchenko, D. Iliadis, N. Ilic, Y. Inamaru, T. Ince, P. Ioannou, M. Iodice, K. Iordanidou, V. Ippolito, A. Irles Quiles, C. Isaksson, M. Ishino, M. Ishitsuka, R. Ishmukhametov, C. Issever, S. Istin, J. M. Iturbe Ponce, R. Iuppa, J. Ivarsson, W. Iwanski, H. Iwasaki, J. M. Izen, V. Izzo, B. Jackson, M. Jackson, P. Jackson, M. R. Jaekel, V. Jain, K. Jakobs, S. Jakobsen, T. Jakoubek, J. Jakubek, D. O. Jamin, D. K. Jana, E. Jansen, H. Jansen, J. Janssen, M. Janus, G. Jarlskog, N. Javadov, T. Javůrek, L. Jeanty, J. Jejelava, G.-Y. Jeng, D. Jennens, P. Jenni, J. Jentzsch, C. Jeske, S. Jézéquel, H. Ji, J. Jia, Y. Jiang, M. Jimenez Belenguer, S. Jin, A. Jinaru, O. Jinnouchi, M. D. Joergensen, K. E. Johansson, P. Johansson, K. A. Johns, K. Jon-And, G. Jones, R. W. L. Jones, T. J. Jones, J. Jongmanns, P. M. Jorge, K. D. Joshi, J. Jovicevic, X. Ju, C. A. Jung, P. Jussel, A. Juste Rozas, M. Kaci, A. Kaczmarska, M. Kado, H. Kagan, M. Kagan, E. Kajomovitz, C. W. Kalderon, S. Kama, A. Kamenshchikov, N. Kanaya, M. Kaneda, S. Kaneti, V. A. Kantserov, J. Kanzaki, B. Kaplan, A. Kapliy, D. Kar, K. Karakostas, A. Karamaoun, N. Karastathis, M. J. Kareem, M. Karnevskiy, S. N. Karpov, Z. M. Karpova, K. Karthik, V. Kartvelishvili, A. N. Karyukhin, L. Kashif, G. Kasieczka, R. D. Kass, A. Kastanas, Y. Kataoka, A. Katre, J. Katzy, V. Kaushik, K. Kawagoe, T. Kawamoto, G. Kawamura, S. Kazama, V. F. Kazanin, M. Y. Kazarinov, R. Keeler, R. Kehoe, M. Keil, J. S. Keller, J. J. Kempster, H. Keoshkerian, O. Kepka, B. P. Kerševan, S. Kersten, K. Kessoku, J. Keung, R. A. Keyes, F. Khalil-zada, H. Khandanyan, A. Khanov, A. Kharlamov, A. Khodinov, A. Khomich, T. J. Khoo, G. Khoriauli, V. Khovanskiy, E. Khramov, J. Khubua, H. Y. Kim, H. Kim, S. H. Kim, N. Kimura, O. Kind, B. T. King, M. King, R. S. B. King, S. B. King, J. Kirk, A. E. Kiryunin, T. Kishimoto, D. Kisielewska, F. Kiss, K. Kiuchi, E. Kladiva, M. Klein, U. Klein, K. Kleinknecht, P. Klimek, A. Klimentov, R. Klingenberg, J. A. Klinger, T. Klioutchnikova, P. F. Klok, E.-E. Kluge, P. Kluit, S. Kluth, E. Kneringer, E. B. F. G. Knoops, A. Knue, D. Kobayashi, T. Kobayashi, M. Kobel, M. Kocian, P. Kodys, T. Koffas, E. Koffeman, L. A. Kogan, S. Kohlmann, Z. Kohout, T. Kohriki, T. Koi, H. Kolanoski, I. Koletsou, J. Koll, A. A. Komar, Y. Komori, T. Kondo, N. Kondrashova, K. Köneke, A. C. König, S. König, T. Kono, R. Konoplich, N. Konstantinidis, R. Kopeliansky, S. Koperny, L. Köpke, A. K. Kopp, K. Korcyl, K. Kordas, A. Korn, A. A. Korol, I. Korolkov, E. V. Korolkova, V. A. Korotkov, O. Kortner, S. Kortner, V. V. Kostyukhin, V. M. Kotov, A. Kotwal, A. Kourkoumeli-Charalampidi, C. Kourkoumelis, V. Kouskoura, A. Koutsman, R. Kowalewski, T. Z. Kowalski, W. Kozanecki, A. S. Kozhin, V. A. Kramarenko, G. Kramberger, D. Krasnopevtsev, M. W. Krasny, A. Krasznahorkay, J. K. Kraus, A. Kravchenko, S. Kreiss, M. Kretz, J. Kretzschmar, K. Kreutzfeldt, P. Krieger, K. Kroeninger, H. Kroha, J. Kroll, J. Kroseberg, J. Krstic, U. Kruchonak, H. Krüger, T. Kruker, N. Krumnack, Z. V. Krumshteyn, A. Kruse, M. C. Kruse, M. Kruskal, T. Kubota, H. Kucuk, S. Kuday, S. Kuehn, A. Kugel, A. Kuhl, T. Kuhl, V. Kukhtin, Y. Kulchitsky, S. Kuleshov, M. Kuna, T. Kunigo, A. Kupco, H. Kurashige, Y. A. Kurochkin, R. Kurumida, V. Kus, E. S. Kuwertz, M. Kuze, J. Kvita, D. Kyriazopoulos, A. La Rosa, L. La Rotonda, C. Lacasta, F. Lacava, J. Lacey, H. Lacker, D. Lacour, V. R. Lacuesta, E. Ladygin, R. Lafaye, B. Laforge, T. Lagouri, S. Lai, H. Laier, L. Lambourne, S. Lammers, C. L. Lampen, W. Lampl, E. Lançon, U. Landgraf, M. P. J. Landon, V. S. Lang, A. J. Lankford, F. Lanni, K. Lantzsch, S. Laplace, C. Lapoire, J. F. Laporte, T. Lari, F. Lasagni Manghi, M. Lassnig, P. Laurelli, W. Lavrijsen, A. T. Law, P. Laycock, O. Le Dortz, E. Le Guirriec, E. Le Menedeu, T. LeCompte, F. Ledroit-Guillon, C. A. Lee, H. Lee, S. C. Lee, L. Lee, G. Lefebvre, M. Lefebvre, F. Legger, C. Leggett, A. Lehan, G. Lehmann Miotto, X. Lei, W. A. Leight, A. Leisos, A. G. Leister, M. A. L. Leite, R. Leitner, D. Lellouch, B. Lemmer, K. J. C. Leney, T. Lenz, G. Lenzen, B. Lenzi, R. Leone, S. Leone, C. Leonidopoulos, S. Leontsinis, C. Leroy, C. G. Lester, C. M. Lester, M. Levchenko, J. Levêque, D. Levin, L. J. Levinson, M. Levy, A. Lewis, G. H. Lewis, A. M. Leyko, M. Leyton, B. Li, B. Li, H. Li, H. L. Li, L. Li, L. Li, S. Li, Y. Li, Z. Liang, H. Liao, B. Liberti, P. Lichard, K. Lie, J. Liebal, W. Liebig, C. Limbach, A. Limosani, S. C. Lin, T. H. Lin, F. Linde, B. E. Lindquist, J. T. Linnemann, E. Lipeles, A. Lipniacka, M. Lisovyi, T. M. Liss, D. Lissauer, A. Lister, A. M. Litke, B. Liu, D. Liu, J. B. Liu, K. Liu, L. Liu, M. Liu, M. Liu, Y. Liu, M. Livan, A. Lleres, J. Llorente Merino, S. L. Lloyd, F. Lo Sterzo, E. Lobodzinska, P. Loch, W. S. Lockman, F. K. Loebinger, A. E. Loevschall-Jensen, A. Loginov, T. Lohse, K. Lohwasser, M. Lokajicek, V. P. Lombardo, B. A. Long, J. D. Long, R. E. Long, L. Lopes, D. Lopez Mateos, B. Lopez Paredes, I. Lopez Paz, J. Lorenz, N. Lorenzo Martinez, M. Losada, P. Loscutoff, X. Lou, A. Lounis, J. Love, P. A. Love, A. J. Lowe, F. Lu, N. Lu, H. J. Lubatti, C. Luci, A. Lucotte, F. Luehring, W. Lukas, L. Luminari, O. Lundberg, B. Lund-Jensen, M. Lungwitz, D. Lynn, R. Lysak, E. Lytken, H. Ma, L. L. Ma, G. Maccarrone, A. Macchiolo, J. Machado Miguens, D. Macina, D. Madaffari, R. Madar, H. J. Maddocks, W. F. Mader, A. Madsen, M. Maeno, T. Maeno, A. Maevskiy, E. Magradze, K. Mahboubi, J. Mahlstedt, S. Mahmoud, C. Maiani, C. Maidantchik, A. A. Maier, A. Maio, S. Majewski, Y. Makida, N. Makovec, P. Mal, B. Malaescu, Pa. Malecki, V. P. Maleev, F. Malek, U. Mallik, D. Malon, C. Malone, S. Maltezos, V. M. Malyshev, S. Malyukov, J. Mamuzic, B. Mandelli, L. Mandelli, I. Mandić, R. Mandrysch, J. Maneira, A. Manfredini, L. Manhaes de Andrade Filho, J. A. Manjarres Ramos, A. Mann, P. M. Manning, A. Manousakis-Katsikakis, B. Mansoulie, R. Mantifel, L. Mapelli, L. March, J. F. Marchand, G. Marchiori, M. Marcisovsky, C. P. Marino, M. Marjanovic, F. Marroquim, S. P. Marsden, Z. Marshall, L. F. Marti, S. Marti-Garcia, B. Martin, B. Martin, T. A. Martin, V. J. Martin, B. Martin dit Latour, H. Martinez, M. Martinez, S. Martin-Haugh, A. C. Martyniuk, M. Marx, F. Marzano, A. Marzin, L. Masetti, T. Mashimo, R. Mashinistov, J. Masik, A. L. Maslennikov, I. Massa, L. Massa, N. Massol, P. Mastrandrea, A. Mastroberardino, T. Masubuchi, P. Mättig, J. Mattmann, J. Maurer, S. J. Maxfield, D. A. Maximov, R. Mazini, L. Mazzaferro, G. Mc Goldrick, S. P. Mc Kee, A. McCarn, R. L. McCarthy, T. G. McCarthy, N. A. McCubbin, K. W. McFarlane, J. A. Mcfayden, G. Mchedlidze, S. J. McMahon, R. A. McPherson, J. Mechnich, M. Medinnis, S. Meehan, S. Mehlhase, A. Mehta, K. Meier, C. Meineck, B. Meirose, C. Melachrinos, B. R. Mellado Garcia, F. Meloni, A. Mengarelli, S. Menke, E. Meoni, K. M. Mercurio, S. Mergelmeyer, N. Meric, P. Mermod, L. Merola, C. Meroni, F. S. Merritt, H. Merritt, A. Messina, J. Metcalfe, A. S. Mete, C. Meyer, C. Meyer, J-P. Meyer, J. Meyer, R. P. Middleton, S. Migas, S. Miglioranzi, L. Mijović, G. Mikenberg, M. Mikestikova, M. Mikuž, A. Milic, D. W. Miller, C. Mills, A. Milov, D. A. Milstead, A. A. Minaenko, Y. Minami, I. A. Minashvili, A. I. Mincer, B. Mindur, M. Mineev, Y. Ming, L. M. Mir, G. Mirabelli, T. Mitani, J. Mitrevski, V. A. Mitsou, A. Miucci, P. S. Miyagawa, J. U. Mjörnmark, T. Moa, K. Mochizuki, S. Mohapatra, W. Mohr, S. Molander, R. Moles-Valls, K. Mönig, C. Monini, J. Monk, E. Monnier, J. Montejo Berlingen, F. Monticelli, S. Monzani, R. W. Moore, N. Morange, D. Moreno, M. Moreno Llácer, P. Morettini, M. Morgenstern, M. Morii, V. Morisbak, S. Moritz, A. K. Morley, G. Mornacchi, J. D. Morris, A. Morton, L. Morvaj, H. G. Moser, M. Mosidze, J. Moss, K. Motohashi, R. Mount, E. Mountricha, S. V. Mouraviev, E. J. W. Moyse, S. Muanza, R. D. Mudd, F. Mueller, J. Mueller, K. Mueller, T. Mueller, T. Mueller, D. Muenstermann, Y. Munwes, J. A. Murillo Quijada, W. J. Murray, H. Musheghyan, E. Musto, A. G. Myagkov, M. Myska, O. Nackenhorst, J. Nadal, K. Nagai, R. Nagai, Y. Nagai, K. Nagano, A. Nagarkar, Y. Nagasaka, K. Nagata, M. Nagel, A. M. Nairz, Y. Nakahama, K. Nakamura, T. Nakamura, I. Nakano, H. Namasivayam, G. Nanava, R. F. Naranjo Garcia, R. Narayan, T. Nattermann, T. Naumann, G. Navarro, R. Nayyar, H. A. Neal, P. Yu. Nechaeva, T. J. Neep, P. D. Nef, A. Negri, G. Negri, M. Negrini, S. Nektarijevic, C. Nellist, A. Nelson, T. K. Nelson, S. Nemecek, P. Nemethy, A. A. Nepomuceno, M. Nessi, M. S. Neubauer, M. Neumann, R. M. Neves, P. Nevski, P. R. Newman, D. H. Nguyen, R. B. Nickerson, R. Nicolaidou, B. Nicquevert, J. Nielsen, N. Nikiforou, A. Nikiforov, V. Nikolaenko, I. Nikolic-Audit, K. Nikolics, K. Nikolopoulos, P. Nilsson, Y. Ninomiya, A. Nisati, R. Nisius, T. Nobe, L. Nodulman, M. Nomachi, I. Nomidis, S. Norberg, M. Nordberg, O. Novgorodova, S. Nowak, M. Nozaki, L. Nozka, K. Ntekas, G. Nunes Hanninger, T. Nunnemann, E. Nurse, F. Nuti, B. J. O’Brien, F. O’grady, D. C. O’Neil, V. O’Shea, F. G. Oakham, H. Oberlack, T. Obermann, J. Ocariz, A. Ochi, I. Ochoa, S. Oda, S. Odaka, H. Ogren, A. Oh, S. H. Oh, C. C. Ohm, H. Ohman, H. Oide, W. Okamura, H. Okawa, Y. Okumura, T. Okuyama, A. Olariu, A. G. Olchevski, S. A. Olivares Pino, D. Oliveira Damazio, E. Oliver Garcia, A. Olszewski, J. Olszowska, A. Onofre, P. U. E. Onyisi, C. J. Oram, M. J. Oreglia, Y. Oren, D. Orestano, N. Orlando, C. Oropeza Barrera, R. S. Orr, B. Osculati, R. Ospanov, G. Otero y Garzon, H. Otono, M. Ouchrif, E. A. Ouellette, F. Ould-Saada, A. Ouraou, K. P. Oussoren, Q. Ouyang, A. Ovcharova, M. Owen, V. E. Ozcan, N. Ozturk, K. Pachal, A. Pacheco Pages, C. Padilla Aranda, M. Pagáčová, S. Pagan Griso, E. Paganis, C. Pahl, F. Paige, P. Pais, K. Pajchel, G. Palacino, S. Palestini, M. Palka, D. Pallin, A. Palma, J. D. Palmer, Y. B. Pan, E. Panagiotopoulou, J. G. Panduro Vazquez, P. Pani, N. Panikashvili, S. Panitkin, D. Pantea, L. Paolozzi, Th. D. Papadopoulou, K. Papageorgiou, A. Paramonov, D. Paredes Hernandez, M. A. Parker, F. Parodi, J. A. Parsons, U. Parzefall, E. Pasqualucci, S. Passaggio, A. Passeri, F. Pastore, Fr. Pastore, G. Pásztor, S. Pataraia, N. D. Patel, J. R. Pater, S. Patricelli, T. Pauly, J. Pearce, L. E. Pedersen, M. Pedersen, S. Pedraza Lopez, R. Pedro, S. V. Peleganchuk, D. Pelikan, H. Peng, B. Penning, J. Penwell, D. V. Perepelitsa, E. Perez Codina, M. T. Pérez García-Estañ, L. Perini, H. Pernegger, S. Perrella, R. Perrino, R. Peschke, V. D. Peshekhonov, K. Peters, R. F. Y. Peters, B. A. Petersen, T. C. Petersen, E. Petit, A. Petridis, C. Petridou, E. Petrolo, F. Petrucci, N. E. Pettersson, R. Pezoa, P. W. Phillips, G. Piacquadio, E. Pianori, A. Picazio, E. Piccaro, M. Piccinini, M. A. Pickering, R. Piegaia, D. T. Pignotti, J. E. Pilcher, A. D. Pilkington, J. Pina, M. Pinamonti, A. Pinder, J. L. Pinfold, A. Pingel, B. Pinto, S. Pires, M. Pitt, C. Pizio, L. Plazak, M.-A. Pleier, V. Pleskot, E. Plotnikova, P. Plucinski, D. Pluth, S. Poddar, F. Podlyski, R. Poettgen, L. Poggioli, D. Pohl, M. Pohl, G. Polesello, A. Policicchio, R. Polifka, A. Polini, C. S. Pollard, V. Polychronakos, K. Pommès, L. Pontecorvo, B. G. Pope, G. A. Popeneciu, D. S. Popovic, A. Poppleton, X. Portell Bueso, S. Pospisil, K. Potamianos, I. N. Potrap, C. J. Potter, C. T. Potter, G. Poulard, J. Poveda, V. Pozdnyakov, P. Pralavorio, A. Pranko, S. Prasad, R. Pravahan, S. Prell, D. Price, J. Price, L. E. Price, D. Prieur, M. Primavera, M. Proissl, K. Prokofiev, F. Prokoshin, E. Protopapadaki, S. Protopopescu, J. Proudfoot, M. Przybycien, H. Przysiezniak, E. Ptacek, D. Puddu, E. Pueschel, D. Puldon, M. Purohit, P. Puzo, J. Qian, G. Qin, Y. Qin, A. Quadt, D. R. Quarrie, W. B. Quayle, M. Queitsch-Maitland, D. Quilty, A. Qureshi, V. Radeka, V. Radescu, S. K. Radhakrishnan, P. Radloff, P. Rados, F. Ragusa, G. Rahal, S. Rajagopalan, M. Rammensee, C. Rangel-Smith, K. Rao, F. Rauscher, T. C. Rave, T. Ravenscroft, M. Raymond, A. L. Read, N. P. Readioff, D. M. Rebuzzi, A. Redelbach, G. Redlinger, R. Reece, K. Reeves, L. Rehnisch, H. Reisin, M. Relich, C. Rembser, H. Ren, Z. L. Ren, A. Renaud, M. Rescigno, S. Resconi, O. L. Rezanova, P. Reznicek, R. Rezvani, R. Richter, M. Ridel, P. Rieck, J. Rieger, M. Rijssenbeek, A. Rimoldi, L. Rinaldi, E. Ritsch, I. Riu, F. Rizatdinova, E. Rizvi, S. H. Robertson, A. Robichaud-Veronneau, D. Robinson, J. E. M. Robinson, A. Robson, C. Roda, L. Rodrigues, S. Roe, O. Røhne, S. Rolli, A. Romaniouk, M. Romano, E. Romero Adam, N. Rompotis, M. Ronzani, L. Roos, E. Ros, S. Rosati, K. Rosbach, M. Rose, P. Rose, P. L. Rosendahl, O. Rosenthal, V. Rossetti, E. Rossi, L. P. Rossi, R. Rosten, M. Rotaru, I. Roth, J. Rothberg, D. Rousseau, C. R. Royon, A. Rozanov, Y. Rozen, X. Ruan, F. Rubbo, I. Rubinskiy, V. I. Rud, C. Rudolph, M. S. Rudolph, F. Rühr, A. Ruiz-Martinez, Z. Rurikova, N. A. Rusakovich, A. Ruschke, H. L. Russell, J. P. Rutherfoord, N. Ruthmann, Y. F. Ryabov, M. Rybar, G. Rybkin, N. C. Ryder, A. F. Saavedra, G. Sabato, S. Sacerdoti, A. Saddique, I. Sadeh, H. F-W. Sadrozinski, R. Sadykov, F. Safai Tehrani, H. Sakamoto, Y. Sakurai, G. Salamanna, A. Salamon, M. Saleem, D. Salek, P. H. Sales De Bruin, D. Salihagic, A. Salnikov, J. Salt, D. Salvatore, F. Salvatore, A. Salvucci, A. Salzburger, D. Sampsonidis, A. Sanchez, J. Sánchez, V. Sanchez Martinez, H. Sandaker, R. L. Sandbach, H. G. Sander, M. P. Sanders, M. Sandhoff, T. Sandoval, C. Sandoval, R. Sandstroem, D. P. C. Sankey, A. Sansoni, C. Santoni, R. Santonico, H. Santos, I. Santoyo Castillo, K. Sapp, A. Sapronov, J. G. Saraiva, B. Sarrazin, G. Sartisohn, O. Sasaki, Y. Sasaki, G. Sauvage, E. Sauvan, P. Savard, D. O. Savu, C. Sawyer, L. Sawyer, D. H. Saxon, J. Saxon, C. Sbarra, A. Sbrizzi, T. Scanlon, D. A. Scannicchio, M. Scarcella, V. Scarfone, J. Schaarschmidt, P. Schacht, D. Schaefer, R. Schaefer, S. Schaepe, S. Schaetzel, U. Schäfer, A. C. Schaffer, D. Schaile, R. D. Schamberger, V. Scharf, V. A. Schegelsky, D. Scheirich, M. Schernau, M. I. Scherzer, C. Schiavi, J. Schieck, C. Schillo, M. Schioppa, S. Schlenker, E. Schmidt, K. Schmieden, C. Schmitt, S. Schmitt, B. Schneider, Y. J. Schnellbach, U. Schnoor, L. Schoeffel, A. Schoening, B. D. Schoenrock, A. L. S. Schorlemmer, M. Schott, D. Schouten, J. Schovancova, S. Schramm, M. Schreyer, C. Schroeder, N. Schuh, M. J. Schultens, H.-C. Schultz-Coulon, H. Schulz, M. Schumacher, B. A. Schumm, Ph. Schune, C. Schwanenberger, A. Schwartzman, T. A. Schwarz, Ph. Schwegler, Ph. Schwemling, R. Schwienhorst, J. Schwindling, T. Schwindt, M. Schwoerer, F. G. Sciacca, E. Scifo, G. Sciolla, F. Scuri, F. Scutti, J. Searcy, G. Sedov, E. Sedykh, P. Seema, S. C. Seidel, A. Seiden, F. Seifert, J. M. Seixas, G. Sekhniaidze, S. J. Sekula, K. E. Selbach, D. M. Seliverstov, G. Sellers, N. Semprini-Cesari, C. Serfon, L. Serin, L. Serkin, T. Serre, R. Seuster, H. Severini, T. Sfiligoj, F. Sforza, A. Sfyrla, E. Shabalina, M. Shamim, L. Y. Shan, R. Shang, J. T. Shank, M. Shapiro, P. B. Shatalov, K. Shaw, A. Shcherbakova, C. Y. Shehu, P. Sherwood, L. Shi, S. Shimizu, C. O. Shimmin, M. Shimojima, M. Shiyakova, A. Shmeleva, D. Shoaleh Saadi, M. J. Shochet, D. Short, S. Shrestha, E. Shulga, M. A. Shupe, S. Shushkevich, P. Sicho, O. Sidiropoulou, D. Sidorov, A. Sidoti, F. Siegert, Dj. Sijacki, J. Silva, Y. Silver, D. Silverstein, S. B. Silverstein, V. Simak, O. Simard, Lj. Simic, S. Simion, E. Simioni, B. Simmons, D. Simon, R. Simoniello, P. Sinervo, N. B. Sinev, G. Siragusa, A. Sircar, A. N. Sisakyan, S. Yu. Sivoklokov, J. Sjölin, T. B. Sjursen, H. P. Skottowe, P. Skubic, M. Slater, T. Slavicek, M. Slawinska, K. Sliwa, V. Smakhtin, B. H. Smart, L. Smestad, S. Yu. Smirnov, Y. Smirnov, L. N. Smirnova, O. Smirnova, K. M. Smith, M. Smizanska, K. Smolek, A. A. Snesarev, G. Snidero, S. Snyder, R. Sobie, F. Socher, A. Soffer, D. A. Soh, C. A. Solans, M. Solar, J. Solc, E. Yu. Soldatov, U. Soldevila, A. A. Solodkov, A. Soloshenko, O. V. Solovyanov, V. Solovyev, P. Sommer, H. Y. Song, N. Soni, A. Sood, A. Sopczak, B. Sopko, V. Sopko, V. Sorin, M. Sosebee, R. Soualah, P. Soueid, A. M. Soukharev, D. South, S. Spagnolo, F. Spanò, W. R. Spearman, F. Spettel, R. Spighi, G. Spigo, L. A. Spiller, M. Spousta, T. Spreitzer, R. D. St. Denis, S. Staerz, J. Stahlman, R. Stamen, S. Stamm, E. Stanecka, R. W. Stanek, C. Stanescu, M. Stanescu-Bellu, M. M. Stanitzki, S. Stapnes, E. A. Starchenko, J. Stark, P. Staroba, P. Starovoitov, R. Staszewski, P. Stavina, P. Steinberg, B. Stelzer, H. J. Stelzer, O. Stelzer-Chilton, H. Stenzel, S. Stern, G. A. Stewart, J. A. Stillings, M. C. Stockton, M. Stoebe, G. Stoicea, P. Stolte, S. Stonjek, A. R. Stradling, A. Straessner, M. E. Stramaglia, J. Strandberg, S. Strandberg, A. Strandlie, E. Strauss, M. Strauss, P. Strizenec, R. Ströhmer, D. M. Strom, R. Stroynowski, A. Strubig, S. A. Stucci, B. Stugu, N. A. Styles, D. Su, J. Su, R. Subramaniam, A. Succurro, Y. Sugaya, C. Suhr, M. Suk, V. V. Sulin, S. Sultansoy, T. Sumida, S. Sun, X. Sun, J. E. Sundermann, K. Suruliz, G. Susinno, M. R. Sutton, Y. Suzuki, M. Svatos, S. Swedish, M. Swiatlowski, I. Sykora, T. Sykora, D. Ta, C. Taccini, K. Tackmann, J. Taenzer, A. Taffard, R. Tafirout, N. Taiblum, H. Takai, R. Takashima, H. Takeda, T. Takeshita, Y. Takubo, M. Talby, A. A. Talyshev, J. Y. C. Tam, K. G. Tan, J. Tanaka, R. Tanaka, S. Tanaka, S. Tanaka, A. J. Tanasijczuk, B. B. Tannenwald, N. Tannoury, S. Tapprogge, S. Tarem, F. Tarrade, G. F. Tartarelli, P. Tas, M. Tasevsky, T. Tashiro, E. Tassi, A. Tavares Delgado, Y. Tayalati, F. E. Taylor, G. N. Taylor, W. Taylor, F. A. Teischinger, M. Teixeira Dias Castanheira, P. Teixeira-Dias, K. K. Temming, H. Ten Kate, P. K. Teng, J. J. Teoh, S. Terada, K. Terashi, J. Terron, S. Terzo, M. Testa, R. J. Teuscher, J. Therhaag, T. Theveneaux-Pelzer, J. P. Thomas, J. Thomas-Wilsker, E. N. Thompson, P. D. Thompson, P. D. Thompson, R. J. Thompson, A. S. Thompson, L. A. Thomsen, E. Thomson, M. Thomson, W. M. Thong, R. P. Thun, F. Tian, M. J. Tibbetts, V. O. Tikhomirov, Yu. A. Tikhonov, S. Timoshenko, E. Tiouchichine, P. Tipton, S. Tisserant, T. Todorov, S. Todorova-Nova, J. Tojo, S. Tokár, K. Tokushuku, K. Tollefson, E. Tolley, L. Tomlinson, M. Tomoto, L. Tompkins, K. Toms, N. D. Topilin, E. Torrence, H. Torres, E. Torró Pastor, J. Toth, F. Touchard, D. R. Tovey, H. L. Tran, T. Trefzger, L. Tremblet, A. Tricoli, I. M. Trigger, S. Trincaz-Duvoid, M. F. Tripiana, W. Trischuk, B. Trocmé, C. Troncon, M. Trottier-McDonald, M. Trovatelli, P. True, M. Trzebinski, A. Trzupek, C. Tsarouchas, J. C-L. Tseng, P. V. Tsiareshka, D. Tsionou, G. Tsipolitis, N. Tsirintanis, S. Tsiskaridze, V. Tsiskaridze, E. G. Tskhadadze, I. I. Tsukerman, V. Tsulaia, S. Tsuno, D. Tsybychev, A. Tudorache, V. Tudorache, A. N. Tuna, S. A. Tupputi, S. Turchikhin, D. Turecek, I. Turk Cakir, R. Turra, A. J. Turvey, P. M. Tuts, A. Tykhonov, M. Tylmad, M. Tyndel, K. Uchida, I. Ueda, R. Ueno, M. Ughetto, M. Ugland, M. Uhlenbrock, F. Ukegawa, G. Unal, A. Undrus, G. Unel, F. C. Ungaro, Y. Unno, C. Unverdorben, J. Urban, D. Urbaniec, P. Urquijo, G. Usai, A. Usanova, L. Vacavant, V. Vacek, B. Vachon, N. Valencic, S. Valentinetti, A. Valero, L. Valery, S. Valkar, E. Valladolid Gallego, S. Vallecorsa, J. A. Valls Ferrer, W. Van Den Wollenberg, P. C. Van Der Deijl, R. van der Geer, H. van der Graaf, R. Van Der Leeuw, D. van der Ster, N. van Eldik, P. van Gemmeren, J. Van Nieuwkoop, I. van Vulpen, M. C. van Woerden, M. Vanadia, W. Vandelli, R. Vanguri, A. Vaniachine, P. Vankov, F. Vannucci, G. Vardanyan, R. Vari, E. W. Varnes, T. Varol, D. Varouchas, A. Vartapetian, K. E. Varvell, F. Vazeille, T. Vazquez Schroeder, J. Veatch, F. Veloso, T. Velz, S. Veneziano, A. Ventura, D. Ventura, M. Venturi, N. Venturi, A. Venturini, V. Vercesi, M. Verducci, W. Verkerke, J. C. Vermeulen, A. Vest, M. C. Vetterli, O. Viazlo, I. Vichou, T. Vickey, O. E. Vickey Boeriu, G. H. A. Viehhauser, S. Viel, R. Vigne, M. Villa, M. Villaplana Perez, E. Vilucchi, M. G. Vincter, V. B. Vinogradov, J. Virzi, I. Vivarelli, F. Vives Vaque, S. Vlachos, D. Vladoiu, M. Vlasak, A. Vogel, M. Vogel, P. Vokac, G. Volpi, M. Volpi, H. von der Schmitt, H. von Radziewski, E. von Toerne, V. Vorobel, K. Vorobev, M. Vos, R. Voss, J. H. Vossebeld, N. Vranjes, M. Vranjes Milosavljevic, V. Vrba, M. Vreeswijk, T. Vu Anh, R. Vuillermet, I. Vukotic, Z. Vykydal, P. Wagner, W. Wagner, H. Wahlberg, S. Wahrmund, J. Wakabayashi, J. Walder, R. Walker, W. Walkowiak, R. Wall, P. Waller, B. Walsh, C. Wang, C. Wang, F. Wang, H. Wang, H. Wang, J. Wang, J. Wang, K. Wang, R. Wang, S. M. Wang, T. Wang, X. Wang, C. Wanotayaroj, A. Warburton, C. P. Ward, D. R. Wardrope, M. Warsinsky, A. Washbrook, C. Wasicki, P. M. Watkins, A. T. Watson, I. J. Watson, M. F. Watson, G. Watts, S. Watts, B. M. Waugh, S. Webb, M. S. Weber, S. W. Weber, J. S. Webster, A. R. Weidberg, B. Weinert, J. Weingarten, C. Weiser, H. Weits, P. S. Wells, T. Wenaus, D. Wendland, Z. Weng, T. Wengler, S. Wenig, N. Wermes, M. Werner, P. Werner, M. Wessels, J. Wetter, K. Whalen, A. White, M. J. White, R. White, S. White, D. Whiteson, D. Wicke, F. J. Wickens, W. Wiedenmann, M. Wielers, P. Wienemann, C. Wiglesworth, L. A. M. Wiik-Fuchs, P. A. Wijeratne, A. Wildauer, M. A. Wildt, H. G. Wilkens, H. H. Williams, S. Williams, C. Willis, S. Willocq, A. Wilson, J. A. Wilson, I. Wingerter-Seez, F. Winklmeier, B. T. Winter, M. Wittgen, T. Wittig, J. Wittkowski, S. J. Wollstadt, M. W. Wolter, H. Wolters, B. K. Wosiek, J. Wotschack, M. J. Woudstra, K. W. Wozniak, M. Wright, M. Wu, S. L. Wu, X. Wu, Y. Wu, E. Wulf, T. R. Wyatt, B. M. Wynne, S. Xella, M. Xiao, D. Xu, L. Xu, B. Yabsley, S. Yacoob, R. Yakabe, M. Yamada, H. Yamaguchi, Y. Yamaguchi, A. Yamamoto, S. Yamamoto, T. Yamamura, T. Yamanaka, K. Yamauchi, Y. Yamazaki, Z. Yan, H. Yang, H. Yang, Y. Yang, S. Yanush, L. Yao, W-M. Yao, Y. Yasu, E. Yatsenko, K. H. Yau Wong, J. Ye, S. Ye, I. Yeletskikh, A. L. Yen, E. Yildirim, M. Yilmaz, R. Yoosoofmiya, K. Yorita, R. Yoshida, K. Yoshihara, C. Young, C. J. S. Young, S. Youssef, D. R. Yu, J. Yu, J. M. Yu, J. Yu, L. Yuan, A. Yurkewicz, I. Yusuff, B. Zabinski, R. Zaidan, A. M. Zaitsev, A. Zaman, S. Zambito, L. Zanello, D. Zanzi, C. Zeitnitz, M. Zeman, A. Zemla, K. Zengel, O. Zenin, T. Ženiš, D. Zerwas, G. Zevi della Porta, D. Zhang, F. Zhang, H. Zhang, J. Zhang, L. Zhang, R. Zhang, X. Zhang, Z. Zhang, X. Zhao, Y. Zhao, Z. Zhao, A. Zhemchugov, J. Zhong, B. Zhou, L. Zhou, L. Zhou, N. Zhou, C. G. Zhu, H. Zhu, J. Zhu, Y. Zhu, X. Zhuang, K. Zhukov, A. Zibell, D. Zieminska, N. I. Zimine, C. Zimmermann, R. Zimmermann, S. Zimmermann, S. Zimmermann, Z. Zinonos, M. Ziolkowski, G. Zobernig, A. Zoccoli, M. zur Nedden, G. Zurzolo, V. Zutshi, L. Zwalinski

**Affiliations:** 1 Department of Physics, University of Adelaide, Adelaide, Australia; 2 Physics Department, SUNY Albany, Albany, NY USA; 3 Department of Physics, University of Alberta, Edmonton, AB Canada; 4 Department of Physics, Ankara University, Ankara, Turkey; Department of Physics, Gazi University, Ankara, Turkey; Istanbul Aydin University, Istanbul, Turkey; Division of Physics, TOBB University of Economics and Technology, Ankara, Turkey; 5 LAPP, CNRS/IN2P3, Université de Savoie, Annecy-le-Vieux, France; 6 High Energy Physics Division, Argonne National Laboratory, Argonne, IL USA; 7 Department of Physics, University of Arizona, Tucson, AZ USA; 8 Department of Physics, The University of Texas at Arlington, Arlington, TX USA; 9 Physics Department, University of Athens, Athens, Greece; 10 Physics Department, National Technical University of Athens, Zografou, Greece; 11 Institute of Physics, Azerbaijan Academy of Sciences, Baku, Azerbaijan; 12 Institut de Física d’Altes Energies, Departament de Física de la Universitat Autònoma de Barcelona, Barcelona, Spain; 13 Institute of Physics, University of Belgrade, Belgrade, Serbia; Vinca Institute of Nuclear Sciences, University of Belgrade, Belgrade, Serbia; 14 Department for Physics and Technology, University of Bergen, Bergen, Norway; 15 Physics Division, Lawrence Berkeley National Laboratory, University of California, Berkeley, CA USA; 16 Department of Physics, Humboldt University, Berlin, Germany; 17 Laboratory for High Energy Physics, Albert Einstein Center for Fundamental Physics, University of Bern, Bern, Switzerland; 18 School of Physics and Astronomy, University of Birmingham, Birmingham, UK; 19 Department of Physics, Bogazici University, Istanbul, Turkey; Department of Physics, Dogus University, Istanbul, Turkey; Department of Physics Engineering, Gaziantep University, Gaziantep, Turkey; 20 INFN Sezione di Bologna, Bologna, Italy; Dipartimento di Fisica e Astronomia, Università di Bologna, Bologna, Italy; 21 Physikalisches Institut, University of Bonn, Bonn, Germany; 22 Department of Physics, Boston University, Boston, MA USA; 23 Department of Physics, Brandeis University, Waltham, MA USA; 24 Universidade Federal do Rio De Janeiro COPPE/EE/IF, Rio de Janeiro, Brazil; Electrical Circuits Department, Federal University of Juiz de Fora (UFJF), Juiz de Fora, Brazil; Federal University of Sao Joao del Rei (UFSJ), Sao Joao del Rei, Brazil; Instituto de Fisica, Universidade de Sao Paulo, São Paulo, Brazil; 25 Physics Department, Brookhaven National Laboratory, Upton, NY USA; 26 National Institute of Physics and Nuclear Engineering, Bucharest, Romania; Physics Department, National Institute for Research and Development of Isotopic and Molecular Technologies, Cluj Napoca, Romania; University Politehnica Bucharest, Bucharest, Romania; West University in Timisoara, Timisoara, Romania; 27 Departamento de Física, Universidad de Buenos Aires, Buenos Aires, Argentina; 28 Cavendish Laboratory, University of Cambridge, Cambridge, UK; 29 Department of Physics, Carleton University, Ottawa, ON Canada; 30 CERN, Geneva, Switzerland; 31 Enrico Fermi Institute, University of Chicago, Chicago, IL USA; 32 Departamento de Física, Pontificia Universidad Católica de Chile, Santiago, Chile; Departamento de Física, Universidad Técnica Federico Santa María, Valparaiso, Chile; 33 Institute of High Energy Physics, Chinese Academy of Sciences, Beijing, China; Department of Modern Physics, University of Science and Technology of China, Hefei, Anhui, China; Department of Physics, Nanjing University, Nanjing, Jiangsu, China; School of Physics, Shandong University, Jinan, Shandong, China; Physics Department, Shanghai Jiao Tong University, Shanghai, China; Physics Department, Tsinghua University, Beijing, 100084 China; 34 Laboratoire de Physique Corpusculaire, Clermont Université, Université Blaise Pascal, CNRS/IN2P3, Clermont-Ferrand, France; 35 Nevis Laboratory, Columbia University, Irvington, NY USA; 36 Niels Bohr Institute, University of Copenhagen, Kobenhavn, Denmark; 37 INFN Gruppo Collegato di Cosenza, Laboratori Nazionali di Frascati, Frascati, Italy; Dipartimento di Fisica, Università della Calabria, Rende, Italy; 38 Faculty of Physics and Applied Computer Science, AGH University of Science and Technology, Kraków, Poland; Marian Smoluchowski Institute of Physics, Jagiellonian University, Kraków, Poland; 39 The Henryk Niewodniczanski Institute of Nuclear Physics, Polish Academy of Sciences, Kraków, Poland; 40 Physics Department, Southern Methodist University, Dallas, TX USA; 41 Physics Department, University of Texas at Dallas, Richardson, TX USA; 42 DESY, Hamburg and Zeuthen, Germany; 43 Institut für Experimentelle Physik IV, Technische Universität Dortmund, Dortmund, Germany; 44 Institut für Kern- und Teilchenphysik, Technische Universität Dresden, Dresden, Germany; 45 Department of Physics, Duke University, Durham, NC USA; 46 SUPA, School of Physics and Astronomy, University of Edinburgh, Edinburgh, UK; 47 INFN Laboratori Nazionali di Frascati, Frascati, Italy; 48 Fakultät für Mathematik und Physik, Albert-Ludwigs-Universität, Freiburg, Germany; 49 Section de Physique, Université de Genève, Geneva, Switzerland; 50 INFN Sezione di Genova, Genoa, Italy; Dipartimento di Fisica, Università di Genova, Genoa, Italy; 51 E. Andronikashvili Institute of Physics, Iv. Javakhishvili Tbilisi State University, Tbilisi, Georgia; High Energy Physics Institute, Tbilisi State University, Tbilisi, Georgia; 52 II Physikalisches Institut, Justus-Liebig-Universität Giessen, Giessen, Germany; 53 SUPA, School of Physics and Astronomy, University of Glasgow, Glasgow, UK; 54 II Physikalisches Institut, Georg-August-Universität, Göttingen, Germany; 55 Laboratoire de Physique Subatomique et de Cosmologie, Université Grenoble-Alpes, CNRS/IN2P3, Grenoble, France; 56 Department of Physics, Hampton University, Hampton, VA USA; 57 Laboratory for Particle Physics and Cosmology, Harvard University, Cambridge, MA USA; 58 Kirchhoff-Institut für Physik, Ruprecht-Karls-Universität Heidelberg, Heidelberg, Germany; Physikalisches Institut, Ruprecht-Karls-Universität Heidelberg, Heidelberg, Germany; ZITI Institut für technische Informatik, Ruprecht-Karls-Universität Heidelberg, Mannheim, Germany; 59 Faculty of Applied Information Science, Hiroshima Institute of Technology, Hiroshima, Japan; 60 Department of Physics, The Chinese University of Hong Kong, Shatin, NT, Hong Kong; Department of Physics, The University of Hong Kong, Pok Fu Lam, Hong Kong; Department of Physics, The Hong Kong University of Science and Technology, Clear Water Bay, Kowloon, Hong Kong, China; 61 Department of Physics, Indiana University, Bloomington, IN USA; 62 Institut für Astro- und Teilchenphysik, Leopold-Franzens-Universität, Innsbruck, Austria; 63 University of Iowa, Iowa City, IA USA; 64 Department of Physics and Astronomy, Iowa State University, Ames, IA USA; 65 Joint Institute for Nuclear Research, JINR Dubna, Dubna, Russia; 66 KEK, High Energy Accelerator Research Organization, Tsukuba, Japan; 67 Graduate School of Science, Kobe University, Kobe, Japan; 68 Faculty of Science, Kyoto University, Kyoto, Japan; 69 Kyoto University of Education, Kyoto, Japan; 70 Department of Physics, Kyushu University, Fukuoka, Japan; 71 Instituto de Física La Plata, CONICET, Universidad Nacional de La Plata, La Plata, Argentina; 72 Physics Department, Lancaster University, Lancaster, UK; 73 INFN Sezione di Lecce, Lecce, Italy; Dipartimento di Matematica e Fisica, Università del Salento, Lecce, Italy; 74 Oliver Lodge Laboratory, University of Liverpool, Liverpool, UK; 75 Department of Physics, Jožef Stefan Institute, University of Ljubljana, Ljubljana, Slovenia; 76 School of Physics and Astronomy, Queen Mary University of London, London, UK; 77 Department of Physics, Royal Holloway University of London, Surrey, UK; 78 Department of Physics and Astronomy, University College London, London, UK; 79 Louisiana Tech University, Ruston, LA USA; 80 Laboratoire de Physique Nucléaire et de Hautes Energies, UPMC, Université Paris-Diderot, CNRS/IN2P3, Paris, France; 81 Fysiska institutionen, Lunds universitet, Lund, Sweden; 82 Departamento de Fisica Teorica C-15, Universidad Autonoma de Madrid, Madrid, Spain; 83 Institut für Physik, Universität Mainz, Mainz, Germany; 84 School of Physics and Astronomy, University of Manchester, Manchester, UK; 85 CPPM, Aix-Marseille Université, CNRS/IN2P3, Marseille, France; 86 Department of Physics, University of Massachusetts, Amherst, MA USA; 87 Department of Physics, McGill University, Montreal, QC Canada; 88 School of Physics, University of Melbourne, Parkville, VIC Australia; 89 Department of Physics, The University of Michigan, Ann Arbor, MI USA; 90 Department of Physics and Astronomy, Michigan State University, East Lansing, MI USA; 91 INFN Sezione di Milano, Milan, Italy; Dipartimento di Fisica, Università di Milano, Milan, Italy; 92 B.I. Stepanov Institute of Physics, National Academy of Sciences of Belarus, Minsk, Republic of Belarus; 93 National Scientific and Educational Centre for Particle and High Energy Physics, Minsk, Republic of Belarus; 94 Department of Physics, Massachusetts Institute of Technology, Cambridge, MA USA; 95 Group of Particle Physics, University of Montreal, Montreal, QC Canada; 96 P.N. Lebedev Institute of Physics, Academy of Sciences, Moscow, Russia; 97 Institute for Theoretical and Experimental Physics (ITEP), Moscow, Russia; 98 National Research Nuclear University MEPhI, Moscow, Russia; 99 D.V. Skobeltsyn Institute of Nuclear Physics, M.V. Lomonosov Moscow State University, Moscow, Russia; 100 Fakultät für Physik, Ludwig-Maximilians-Universität München, Munich, Germany; 101 Max-Planck-Institut für Physik (Werner-Heisenberg-Institut), Munich, Germany; 102 Nagasaki Institute of Applied Science, Nagasaki, Japan; 103 Graduate School of Science, Kobayashi-Maskawa Institute, Nagoya University, Nagoya, Japan; 104 INFN Sezione di Napoli, Napoli, Italy; Dipartimento di Fisica, Università di Napoli, Naples, Italy; 105 Department of Physics and Astronomy, University of New Mexico, Albuquerque, NM USA; 106 Institute for Mathematics, Astrophysics and Particle Physics, Radboud University Nijmegen/Nikhef, Nijmegen, The Netherlands; 107 Nikhef National Institute for Subatomic Physics, University of Amsterdam, Amsterdam, The Netherlands; 108 Department of Physics, Northern Illinois University, DeKalb, IL USA; 109 Budker Institute of Nuclear Physics, SB RAS, Novosibirsk, Russia; 110 Department of Physics, New York University, New York, NY USA; 111 Ohio State University, Columbus, OH USA; 112 Faculty of Science, Okayama University, Okayama, Japan; 113 Homer L. Dodge Department of Physics and Astronomy, University of Oklahoma, Norman, OK USA; 114 Department of Physics, Oklahoma State University, Stillwater, OK USA; 115 RCPTM, Palacký University, Olomouc, Czech Republic; 116 Center for High Energy Physics, University of Oregon, Eugene, OR USA; 117 LAL, Université Paris-Sud, CNRS/IN2P3, Orsay, France; 118 Graduate School of Science, Osaka University, Osaka, Japan; 119 Department of Physics, University of Oslo, Oslo, Norway; 120 Department of Physics, Oxford University, Oxford, UK; 121 INFN Sezione di Pavia, Pavia, Italy; Dipartimento di Fisica, Università di Pavia, Pavia, Italy; 122 Department of Physics, University of Pennsylvania, Philadelphia, PA USA; 123 Petersburg Nuclear Physics Institute, Gatchina, Russia; 124 INFN Sezione di Pisa, Pisa, Italy; Dipartimento di Fisica E. Fermi, Università di Pisa, Pisa, Italy; 125 Department of Physics and Astronomy, University of Pittsburgh, Pittsburgh, PA USA; 126 Laboratorio de Instrumentacao e Fisica Experimental de Particulas, LIP, Lisbon, Portugal; Faculdade de Ciências, Universidade de Lisboa, Lisbon, Portugal; Department of Physics, University of Coimbra, Coimbra, Portugal; Centro de Física Nuclear da Universidade de Lisboa, Lisbon, Portugal; Departamento de Fisica, Universidade do Minho, Braga, Portugal; Departamento de Fisica Teorica y del Cosmos, CAFPE, Universidad de Granada, Granada, Spain; Dep Fisica, CEFITEC of Faculdade de Ciencias e Tecnologia, Universidade Nova de Lisboa, Caparica, Portugal; 127 Institute of Physics, Academy of Sciences of the Czech Republic, Praha, Czech Republic; 128 Czech Technical University in Prague, Praha, Czech Republic; 129 Faculty of Mathematics and Physics, Charles University in Prague, Praha, Czech Republic; 130 State Research Center Institute for High Energy Physics, Protvino, Russia; 131 Particle Physics Department, Rutherford Appleton Laboratory, Didcot, UK; 132 Ritsumeikan University, Kusatsu, Shiga Japan; 133 INFN Sezione di Roma, Rome, Italy; Dipartimento di Fisica, Sapienza Università di Roma, Rome, Italy; 134 INFN Sezione di Roma Tor Vergata, Rome, Italy; Dipartimento di Fisica, Università di Roma Tor Vergata, Rome, Italy; 135 INFN Sezione di Roma Tre, Rome, Italy; Dipartimento di Matematica e Fisica, Università Roma Tre, Rome, Italy; 136 Faculté des Sciences Ain Chock, Réseau Universitaire de Physique des Hautes Energies, Université Hassan II, Casablanca, Morocco; Centre National de l’Energie des Sciences Techniques Nucleaires, Rabat, Morocco; LPHEA-Marrakech, Faculté des Sciences Semlalia, Université Cadi Ayyad, Marrakech, Morocco; LPTPM, Faculté des Sciences, Université Mohamed Premier, Oujda, Morocco; Faculté des Sciences, Université Mohammed V-Agdal, Rabat, Morocco; 137 DSM/IRFU (Institut de Recherches sur les Lois Fondamentales de l’Univers), CEA Saclay (Commissariat à l’Energie Atomique et aux Energies Alternatives), Gif-sur-Yvette, France; 138 Santa Cruz Institute for Particle Physics, University of California Santa Cruz, Santa Cruz, CA USA; 139 Department of Physics, University of Washington, Seattle, WA USA; 140 Department of Physics and Astronomy, University of Sheffield, Sheffield, UK; 141 Department of Physics, Shinshu University, Nagano, Japan; 142 Fachbereich Physik, Universität Siegen, Siegen, Germany; 143 Department of Physics, Simon Fraser University, Burnaby, BC Canada; 144 SLAC National Accelerator Laboratory, Stanford, CA USA; 145 Faculty of Mathematics, Physics and Informatics, Comenius University, Bratislava, Slovak Republic; Department of Subnuclear Physics, Institute of Experimental Physics of the Slovak Academy of Sciences, Kosice, Slovak Republic; 146 Department of Physics, University of Cape Town, Cape Town, South Africa; Department of Physics, University of Johannesburg, Johannesburg, South Africa; School of Physics, University of the Witwatersrand, Johannesburg, South Africa; 147 Department of Physics, Stockholm University, Stockholm, Sweden; The Oskar Klein Centre, Stockholm, Sweden; 148 Physics Department, Royal Institute of Technology, Stockholm, Sweden; 149 Departments of Physics and Astronomy and Chemistry, Stony Brook University, Stony Brook, NY USA; 150 Department of Physics and Astronomy, University of Sussex, Brighton, UK; 151 School of Physics, University of Sydney, Sydney, Australia; 152 Institute of Physics, Academia Sinica, Taipei, Taiwan; 153 Department of Physics, Technion, Israel Institute of Technology, Haifa, Israel; 154 Raymond and Beverly Sackler School of Physics and Astronomy, Tel Aviv University, Tel Aviv, Israel; 155 Department of Physics, Aristotle University of Thessaloniki, Thessaloniki, Greece; 156 Department of Physics, International Center for Elementary Particle Physics, The University of Tokyo, Tokyo, Japan; 157 Graduate School of Science and Technology, Tokyo Metropolitan University, Tokyo, Japan; 158 Department of Physics, Tokyo Institute of Technology, Tokyo, Japan; 159 Department of Physics, University of Toronto, Toronto, ON Canada; 160 TRIUMF, Vancouver, BC, Canada; Department of Physics and Astronomy, York University, Toronto, ON Canada; 161 Faculty of Pure and Applied Sciences, University of Tsukuba, Tsukuba, Japan; 162 Department of Physics and Astronomy, Tufts University, Medford, MA USA; 163 Centro de Investigaciones, Universidad Antonio Narino, Bogota, Colombia; 164 Department of Physics and Astronomy, University of California Irvine, Irvine, CA USA; 165 INFN Gruppo Collegato di Udine, Sezione di Trieste, Udine, Italy; ICTP, Trieste, Italy; Dipartimento di Chimica, Fisica e Ambiente, Università di Udine, Udine, Italy; 166 Department of Physics, University of Illinois, Urbana, IL USA; 167 Department of Physics and Astronomy, University of Uppsala, Uppsala, Sweden; 168 Departamento de Física Atómica, Molecular y Nuclear and Departamento de Ingeniería Electrónica and Instituto de Microelectrónica de Barcelona (IMB-CNM), Instituto de Física Corpuscular (IFIC), University of Valencia, CSIC, Valencia, Spain; 169 Department of Physics, University of British Columbia, Vancouver, BC Canada; 170 Department of Physics and Astronomy, University of Victoria, Victoria, BC Canada; 171 Department of Physics, University of Warwick, Coventry, UK; 172 Waseda University, Tokyo, Japan; 173 Department of Particle Physics, The Weizmann Institute of Science, Rehovot, Israel; 174 Department of Physics, University of Wisconsin, Madison, WI USA; 175 Fakultät für Physik und Astronomie, Julius-Maximilians-Universität, Würzburg, Germany; 176 Fachbereich C Physik, Bergische Universität Wuppertal, Wuppertal, Germany; 177 Department of Physics, Yale University, New Haven, CT USA; 178 Yerevan Physics Institute, Yerevan, Armenia; 179 Centre de Calcul de l’Institut National de Physique Nucléaire et de Physique des Particules (IN2P3), Villeurbanne, France; 180 CERN, 1211 Geneva 23, Switzerland

## Abstract

This article reports on a search for dark matter pair production in association with bottom or top quarks in $20.3 \mathrm {~fb}^{-1}$ of $pp$ collisions collected at $\sqrt{s} = 8$ TeV by the ATLAS detector at the LHC. Events with large missing transverse momentum are selected when produced in association with high-momentum jets of which one or more are identified as jets containing $b$-quarks. Final states with top quarks are selected by requiring a high jet multiplicity and in some cases a single lepton. The data are found to be consistent with the Standard Model expectations and limits are set on the mass scale of effective field theories that describe scalar and tensor interactions between dark matter and Standard Model particles. Limits on the dark-matter–nucleon cross-section for spin-independent and spin-dependent interactions are also provided. These limits are particularly strong for low-mass dark matter. Using a simplified model, constraints are set on the mass of dark matter and of a coloured mediator suitable to explain a possible signal of annihilating dark matter.

## Introduction

The existence of dark matter (DM) in the Universe is highly motivated by many astrophysical and cosmological observations [1–4]. However, its nature remains a mystery. One of the best motivated candidates for a DM particle is a weakly interacting massive particle (WIMP) [5]. At the Large Hadron Collider (LHC), one can search for DM particles ($\chi$) that are pair produced in $pp$ collisions. These studies are sensitive to low DM masses ($m_{\chi }\le$ 10 GeV), and therefore provide information complementary to direct DM searches, which are most sensitive to larger DM masses  [6–9].

If the particles that mediate the interactions between DM and Standard Model (SM) particles are too heavy to be produced directly in the experiment, their interactions can be described by contact operators in the framework of an effective field theory [10–12]. For each operator considered, the reach is expressed in terms of the effective mass scale of the interaction, $M_*$, and of the $\chi$–nucleon cross-section, $\sigma _{\chi -\mathrm{N}}$, as a function of $m_\chi$.

Since DM particles do not interact in the detector, the main signature of DM pair production at colliders is large missing transverse momentum. Initial-state radiation (ISR) of jets, photons, $Z$, or $W$ bosons, was used to tag DM pair production at colliders in several searches at the Tevatron [13] and the LHC [14–22].

**Fig. 1:**
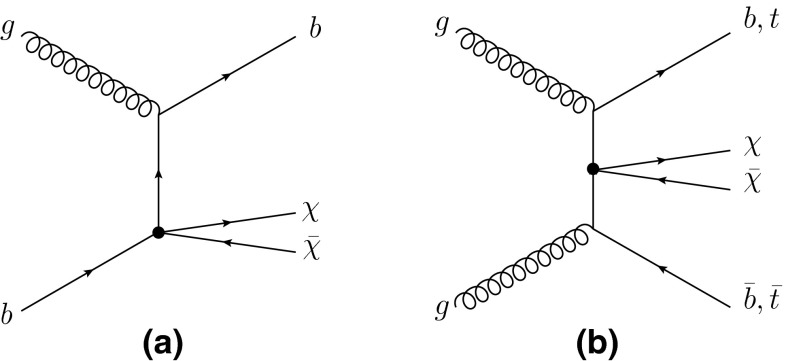
Dominant Feynman diagrams for DM production in conjunction with **a** a single $b$-quark and **b** a heavy quark (*bottom or top*) pair using an effective field theory approach

A new search for DM pair production in association with one $b$-quark or a pair of heavy quarks ($b$ or $t$) was proposed in Ref. [23]. The dominant Feynman diagrams for these processes are shown in Fig. 1. To search for these processes, dedicated selections are defined to reconstruct the various production and decay modes of these heavy-quark final states. For final states containing a semileptonic decay of a top quark, the results of the search for a supersymmetric partner of the top quark are used [24].

**Fig. 2:**
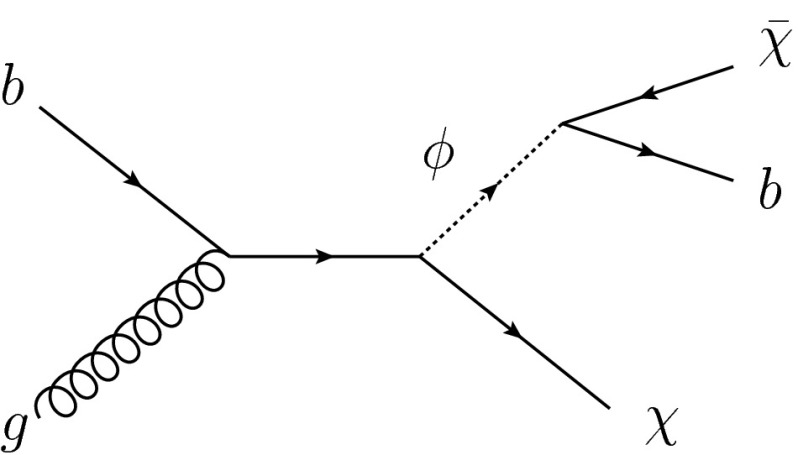
Example of DM production in the $b$-FDM model

The analysis presented in this article is particularly sensitive to effective scalar interactions between DM and quarks described by the operator [12]

1 $$\begin{aligned} \mathcal{O}_\text {scalar}= \sum _q \frac{m_q}{M_*^N}\bar{q}q\bar{\chi }\chi , \end{aligned}$$

where $N=3$ for Dirac DM (D1 operator) and $N=2$ for complex scalar DM (C1 operator). The quark and DM fields are denoted by $q$ and $\chi$, respectively. The scalar operators are normalized by $m_q$, which mitigates contributions to flavour-changing processes, strongly constrained by flavour physics observables [25, 26], through the framework of minimal flavour violation (MFV). The dependence on the quark mass makes final states with bottom and top quarks the most sensitive to these operators.

This search is also sensitive to tensor couplings between DM and quarks. The tensor operator (D9), which describes a magnetic moment coupling, is parameterized as [12]:

2 $$\begin{aligned} \mathcal{O}_\text {tensor} = \sum _q \frac{1}{M_*^{2}}\bar{\chi }\sigma ^{\mu \nu }\chi \bar{q}\sigma _{\mu \nu }q. \end{aligned}$$

MFV suggests that the D9 operator should have a mass dependence from Yukawa couplings although canonically this is not parametrised as such.

The results are also interpreted in light of a bottom-Flavoured Dark Matter model ($b$-FDM) [27]. The $b$-FDM model was proposed to explain the excess of gamma rays from the galactic centre, recently observed by the Fermi Gamma-ray Space Telescope, and interpreted as a signal for DM annihilation [28]. This analysis of the data recorded by the Fermi-LAT collaboration favours DM with a mass of approximately 35 GeV annihilating into $b$-quarks via a coloured mediator. In this model, a new scalar field, $\phi$, mediates the interactions between DM and quarks as shown in Fig. 2. DM is assumed to be a Dirac fermion that couples to right-handed, down-type quarks. The lightest DM particle, which constitutes cosmic DM, preferentially couples to $b$-quarks. The collider signature of this model is $b$-quarks produced in association with missing transverse momentum. This analysis sets constraints on the mass of the mediator and DM particle in the framework of the $b$-FDM model.

**Table 1:** Selections for signal regions 1–4. Variables $p_\mathrm {T}^{j_i}$ ($p_\mathrm {T}^{b_i}$) represent the transverse momentum of the $i$th jet ($b$-tagged jet). The asymmetric transverse mass $am_\mathrm {T2}$ [29–31], $topness$ [32], $m_{jjj}$ and Razor $R$ [33] are used to reject the abundant top quark background

	SR1	SR2	SR3	SR4
Trigger	$E_\mathrm {T}^{\mathrm {miss}}$	$E_\mathrm {T}^{\mathrm {miss}}$	$$5\ $$jets $||\ 4$jets$(1b)$	$E_\mathrm {T}^{\mathrm {miss}}\ ||\ 1$ lepton (no $\tau$)
Jet multiplicity $n_j$	1–2	3–4	$\ge$5	$\ge$4
$b$-Jet multiplicity $n_b$	$>$0 (60 % eff.)	$>$0 (60 % eff.)	$>$1 (70 % eff.)	$>$0 (70 % eff.)
Lepton multiplicity $n_\ell$	0	0	0	1 $\ell$ ($\ell =e, \mu$)
$E_\mathrm {T}^{\mathrm {miss}}$	$>$300 GeV	$>$300 GeV	$>$200 GeV	$>$270 GeV
Jet kinematics	$p_\mathrm {T}^{b_{1}}>100$ GeV	$p_\mathrm {T}^{b_{1}}>100$ GeV	$p_\mathrm {T}^{j}>25$ GeV	$p_\mathrm {T}^{b_{1}}>60$ GeV
		$p_\mathrm {T}^{j_2}>100$ $(60)$ GeV		$p_\mathrm {T}^{1{\text {-}}4}>80, 70, 50, 25$ GeV
Three-jet invariant mass				$m_{jjj}<360$ GeV
$\Delta \phi (j_{i},E_\mathrm {T}^{\mathrm {miss}})$	$>1.0,\ i=1,2$	$>1.0,\ i=1-4$	–	$>0.6,\ i=1,2$
Angular selections	–	–	$\Delta \phi (b_{1},E_\mathrm {T}^{\mathrm {miss}})\ge 1.6$	$\Delta \phi (\ell ,E_\mathrm {T}^{\mathrm {miss}})>0.6$
				$$\Delta R(\ell ,j_{1})<2.75$$
				$$\Delta R(\ell ,b)<3.0$$
Event shape	–	–	Razor $R>$0.75	$topness>2$
$am_\mathrm {T2}$	–	–	–	$>{190}$ GeV
$m_\mathrm{T}^{\ell + E_\mathrm{T}^\mathrm{{miss}}}$	–	–	–	$>{130}$ GeV
$E_\mathrm {T}^{\mathrm {miss}}/ \sqrt{H_\text {T}^{4j}}$	–	–	–	$>{9}$ $\sqrt{\text {GeV}}$

## Detector description and physics objects

The ATLAS detector [34] at the LHC covers the pseudorapidity^1^ range of $| \eta | <4.9$ and is hermetic in azimuth $\phi$. It consists of an inner tracking detector surrounded by a superconducting solenoid, electromagnetic and hadronic calorimeters, and an external muon spectrometer incorporating large superconducting toroidal magnets. A three-level trigger system is used to select events for subsequent offline analysis. The data set used in this analysis consists of $20.3 \mathrm {~fb}^{-1}$ of $pp$ collision data recorded at a centre-of-mass energy of $\sqrt{s} = 8$ TeV with stable beam conditions [35] during the 2012 LHC run. All subsystems listed above were required to be operational.

This analysis requires the reconstruction of muons, electrons, jets, and missing transverse momentum. Muon candidates are identified from tracks that are well reconstructed inside both the inner detector and the muon spectrometer [36]. To reject cosmic-ray muons, muon candidates are required to be consistent with production at the primary vertex, defined as the vertex with the highest $\Sigma (p_\mathrm {T}^{\mathrm {track}} )^2$, where $p_\mathrm {T}^{\mathrm {track}}$ refers to the transverse momentum of each track.

Electrons are identified as tracks that are matched to a well-reconstructed cluster in the electromagnetic calorimeter. Electron candidates must satisfy the *tight* electron shower shape and track selection criteria of Ref. [37]. Both electrons and muons are required to have transverse momenta $p_\mathrm {T}\ > 20$ GeV and $|\eta |<2.5$. Potential ambiguities between overlapping candidate objects are resolved based on their angular separation. If an electron candidate and a jet overlap within $\Delta R< 0.2$, then the object is considered to be an electron and the jet is discarded. If an electron candidate and any jet overlap within $0.2 < \Delta R < 0.4$, or if an electron candidate and a b-tagged jet overlap within $\Delta R< 0.2$ of each other, then the electron is discarded and the jet is retained.

Photon candidates must satisfy the *tight* quality criteria and $|\eta | < 2.37$ [38].

Jet candidates are reconstructed using the anti-$k_t$ clustering algorithm [39] with a radius parameter of 0.4. The inputs to this algorithm are three-dimensional topological clusters [40]. The four-momentum of the jet is defined as the vector sum of the four-momenta of the topological clusters, assuming that each cluster originates from a particle defined to be massless and to come from the interaction point.

To calibrate the reconstructed energy, jets are corrected for the effects of calorimeter response and inhomogeneities using energy- and $\eta$-dependent calibration factors based on simulation and validated with extensive test-beam and collision-data studies [40]. In the simulation, this procedure calibrates the jet energies to those of the corresponding jets constructed from stable simulated particles. In-situ measurements are used to further correct the data to match the energy scale in simulated events. Effects due to additional $pp$ interactions in the same and preceding bunch crossings (pile-up effects) are corrected [41]. Only jets with $p_\mathrm {T}>20 (25)$ GeV and $|\eta |<4.5 (2.5)$ are considered in this analysis for final states involving $b$ ($t$) quarks.

Jets containing particles from the hadronisation of a $b$-quark ($b$-jets) are tagged using a multivariate algorithm [42, 43]. The $b$-tagging algorithm combines the measurement of several quantities distinguishing heavy quarks from light quarks based on their longer lifetime and heavier mass. These quantities include the distance of closest approach of tracks in the jet to the primary event vertex, the number and position of secondary vertices formed by tracks within the jet, as well as the invariant mass associated with such vertices. The algorithm is trained on Monte Carlo (MC) simulations and its performance is calibrated using data. To optimize the sensitivity of this analysis, a requirement on the output of the $b$-tagging algorithm which provides a 60 % (70 %) $b$-jet efficiency operating point is used in signal regions (SR) 1 and 2 (3 and 4) defined below. The corresponding misidentification probability is 15 % (20 %) for $c$-jets, and less than 1 % for light-quark jets. The aforementioned $b$-tagging efficiencies and misidentification probabilities were derived in a simulated $t\bar{t}$ sample with jet transverse momenta of $p_\mathrm {T}>20$ GeV and $|\eta |<2.5$.

The missing transverse momentum, with magnitude $E_\mathrm {T}^{\mathrm {miss}}$, is defined as the negative vector sum of the transverse momenta of jets, muons, electrons, photons, and topological clusters not assigned to any reconstructed objects [44].

**Table 2:** Expected background and signal yields for $m_\chi =10$ GeV compared with observed yields in data for the various signal regions. For the $b$-FDM model, $m_\phi$ is 600 GeV. The row labeled “total expected background” shows the sum of all background components. The quoted uncertainties include all statistical and systematic effects added in quadrature. The effective mass scale, $M_*$, is set to be 100/40/600 GeV for the D1/C1/D9 operators, approximately corresponding to the expected limit. The probabilities of the background-only hypothesis, $p$ values, are also given. The last two lines show the observed and expected 95 % CL upper limits on the number of beyond-the-SM events

Background source	SR1	SR2	SR3	SR4
$Z$($\nu \overline{\nu }$)+jets	190 $\pm$ 26	90 $\pm$ 25	$1^{+6}_{-1}$	–
$W$($\ell \nu$)+jets	133 $\pm$ 23	75 $\pm$ 13		1.3 $\pm$ 0.3
$t\bar{t}$	39 $\pm$ 5	71 $\pm$ 9	87 $\pm$ 11	2.9 $\pm$ 0.6
Single top			8 $\pm$ 3	0.7 $\pm$ 0.3
$t\bar{t}$+$Z$/$W$	–	–	–	1.4 $\pm$ 0.4
Diboson	22 $\pm$ 4	8 $\pm$ 1	–	0.8 $\pm$ 0.4
*Total expected background*	385 $\pm$ 35	245 $\pm$ 30	96 $\pm$ 13	7 $\pm$ 1
*Data*	440	264	107	10
*Expected signal*–D1	10 $\pm$ 2	49 $\pm$ 8	28 $\pm$ 2	35 $\pm$ 5
*Expected signal*–C1	17 $\pm$ 2	61 $\pm$ 9	45 $\pm$ 4	51 $\pm$ 12
*Expected signal*–D9	147 $\pm$ 25	69 $\pm$ 12	2 $\pm$ 1	2 $\pm$ 1
*Expected signal*–$b$-FDM	192 $\pm$ 24	61 $\pm$ 8	1.0 $\pm$ 0.2	–
${ p}$ *value*	0.09	0.29	0.24	0.18
Allowed non SM events–Obs.	124	79	41	10
Allowed non SM events–Exp.	81	67	33	7

## Event selection

Candidate signal events containing at least one high-$p_\mathrm {T}$ jet and large $E_\mathrm {T}^{\mathrm {miss}}$ are assigned to one of four orthogonal signal regions. The first two signal regions focus on events with DM produced in conjunction with one (SR1) or two (SR2) $b$-quarks in the final state. SR3 and SR4 target events in which DM is produced in conjunction with a $t\overline{t}$ pair, where either both top quarks decay hadronically (SR3) or one top quark decays hadronically and the other semileptonically (SR4). SR4 was developed for a top squark search by the ATLAS Collaboration and coincides with the “tNbC_mix” signal region described in Ref. [24]. The four signal regions provide the complementary information needed in case of observation of a signal.

Events assigned to SR1 and SR2 are required to pass a calorimeter-based $E_\mathrm {T}^{\mathrm {miss}}$ trigger with a threshold of 80 GeV. To enrich the sample in $pp \rightarrow \chi \bar{\chi }+b(\bar{b})$, events are required to have a low jet multiplicity ($n_{\text {jets}}<$5), $E_\mathrm {T}^{\mathrm {miss}}$ $>$ 300 GeV, and the most energetic $b$-tagged jet must have a $p_\mathrm {T}$ $>$ 100 GeV. The azimuthal separation between the directions of the jets and the missing transverse momentum is required to be more than 1.0 radian. Events with at least one identified muon or electron are discarded to reject leptonic decays of $W$ and $Z$ bosons. Events satisfying these selection criteria are assigned to SR1 provided that the jet multiplicity does not exceed two. Events are assigned to SR2 when at least three jets are reconstructed in the event and the second most energetic jet has $p_\mathrm {T}> 100$ GeV. If there is a second $b$-tagged jet it has to satisfy $p_\mathrm {T}> 60$ GeV.

Events assigned to SR3 are required to pass triggers specifically designed to select hadronic decays of top quark pairs. Such triggers require either five jets with $p_\mathrm {T}\ge$ 55 GeV each or four jets with $p_\mathrm {T}\ge$ 45 GeV, of which one is tagged as a $b$-jet. To select $pp \rightarrow \chi \bar{\chi }+t\bar{t}$ events, at least five reconstructed jets are required, of which at least two are $b$-tagged, and $E_\mathrm {T}^{\mathrm {miss}}> 200$ GeV. Furthermore, the azimuthal separation between the most energetic $b$-jet and the missing transverse momentum is required to be at least 1.6 radians. To reduce $W/Z$ leptonic decays and leptonic top quark decays, events with at least one identified muon or electron are discarded. To maximize the rejection of the abundant $t\bar{t}$ background, the Razor variable $R$ [33] is used. This variable utilizes both transverse and longitudinal information about the event to fully exploit the kinematics of the decay. To separate signal and background, $R >0.75$ is required.

**Fig. 3:**
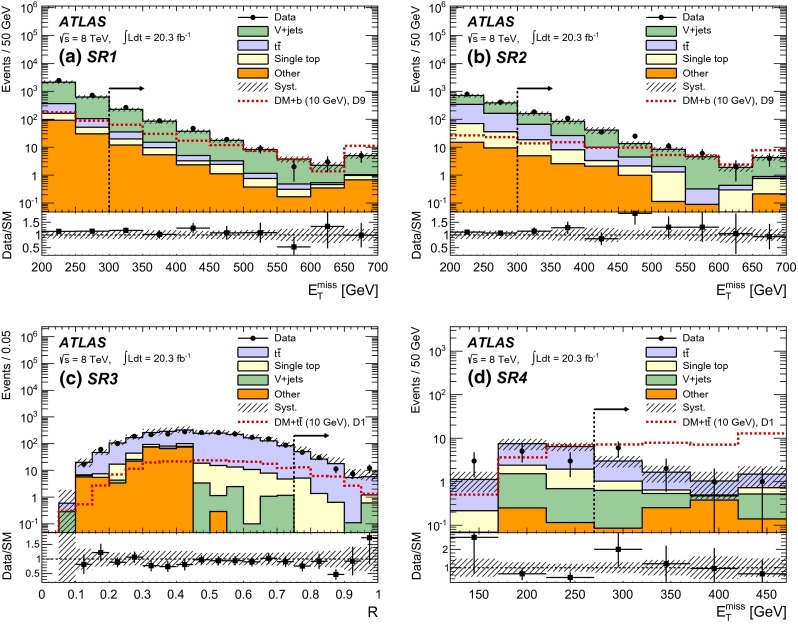
Comparison between data and expected SM background. **a**, **b** $E_\mathrm {T}^{\mathrm {miss}}$ variable for SR1 and SR2 and for an example signal with the operator D9. **c** $R$ variable for SR3 excluding the selection on $R$ and for an example signal with the operator D1. **d** $E_\mathrm {T}^{\mathrm {miss}}$ variable for SR4 excluding the selection on $E_\mathrm {T}^{\mathrm {miss}}$and for an example signal with the operator D1. Other backgrounds are composed of diboson and multijet production. The expected signal for $\chi \bar{\chi } + b(\bar{b})$ (SR1, 2) and for $\chi \bar{\chi } + t\bar{t}$ (SR3, 4) production for $m_\chi =$ 10 GeV is given by the red line assuming $M_*= 100/40/600$ GeV for the D1/C1/D9 operators, respectively. The final selection requirements are indicated by an *arrow*. The *error bars* represent the statistical uncertainty. The *dashed area* shows the systematic uncertainty on the background estimation. Events with values exceeding the range presented are included in the highest bin

To enrich the sample in $pp \rightarrow \chi \bar{\chi }+t\bar{t}$ with one semileptonic decay of the $t$ quark, events assigned to SR4 use single-lepton or $E_\mathrm {T}^{\mathrm {miss}}$ triggers, and require exactly one isolated lepton (electron or muon) with $p_\mathrm {T}> 25$ GeV, at least four high-$p_\mathrm {T}$ jets, where one jet is $b$-tagged with $p_\mathrm {T}> 60$ GeV. Events with $E_\mathrm {T}^{\mathrm {miss}}>270$ GeV are selected when the transverse mass^2^ formed by the lepton and $E_\mathrm {T}^{\mathrm {miss}}$, $m_\mathrm {T}(\ell ,E_\mathrm {T}^{\mathrm {miss}})$, exceeds 130 GeV and $E_\mathrm {T}^{\mathrm {miss}}/ \sqrt{H_\text {T}^{4j}} > 9~\sqrt{\text {GeV}}$, with $H_\text {T}^{4j} = \sum _{i=1}^4 p_\mathrm {T}(\text {jet}_i)$ and where the jets are ordered by decreasing $p_\mathrm {T}$. The azimuthal angle between the missing transverse momentum and the two most energetic jets is required to be greater than 0.6 radians.

Special variables, such as the asymmetric transverse mass $am_\mathrm {T2}$ [29–31] and the $topness$ variable [32], are used to reject the dileptonic $t\bar{t}$ component of the background. Details can be found in Ref. [24]. The diboson background is suppressed by a requirement on the three-jet invariant mass ($m_{jjj}<360$ GeV) [24]. A $\tau$ veto rejects $t\bar{t}$ events with hadronically decaying $\tau$ leptons in the final state. Additional selection criteria [24] on the angles between the lepton and the various jets are imposed to further reduce the $t\bar{t}$ background. Table 1 provides an overview of the selections applied in all four signal regions.

The product of the detector acceptance ${A}$ and the reconstruction efficiency $\epsilon$ for the selections described above varies between 0.1 and 8 % depending on the signal region, operator, and specific channel considered. SR1 and SR2 have the highest efficiencies (${A} \times \epsilon >2\,\%$) for the D9 operator, while SR3 and SR4 are most efficient for the D1 and C1 operators (${A} \times \epsilon >1\,\%$).

The dominant background for SR1 and SR2 is due to $Z\rightarrow \nu \overline{\nu }$ events produced in conjunction with one or more jets. This irreducible background is estimated from data using two control regions (CRs). The first CR exploits $Z+$jets events with $Z\rightarrow \mu ^+\mu ^-$, while the second uses $\gamma +$jets events for which the production at high transverse momentum ($p_\mathrm {T}^{\gamma }>M_Z$) mimics that of $Z+$jets [45]. The $\gamma +$jets control region substantially increases the number of events at large missing transverse momentum. The transverse momentum of the dimuon pair or photon is added vectorially to the $E_\mathrm {T}^{\mathrm {miss}}$ of the event to simulate the $Z\rightarrow \nu \bar{\nu }$ background. Corrections to compensate for the differences in efficiency and acceptance between the $Z(\nu \overline{\nu })+$jets and $Z(\mu ^+\mu ^-)+$jets or $\gamma +$jets are derived from data using control regions without $b$-tagged jets before applying any requirements on the missing transverse momentum. Remaining kinematic selections correspond to the ones described in Table 1. A muon control region is chosen because the energy loss of muons in the detector is comparatively small. The systematic uncertainties introduced by this data-driven procedure on the $Z(\nu \bar{\nu })$+jets background are approximately $10$ %, mainly from the flavour composition of background processes, kinematic differences between the control and signal regions and relative normalizations of backgrounds.

Production of $W/Z+$jets with subsequent leptonic decays of $W$ and to a much smaller degree $Z$ is also a substantial source of background for SR1 and SR2 when the resulting charged leptons fail to be identified or if the $W$ or $Z$ bosons decay to $\tau$ leptons. These contributions are estimated from $Z(\ell ^+ \ell ^-)+$jets and $W(\ell \nu )+$jets MC samples generated using ALPGEN2.3 [46] with the CTEQ6L1 [47] parton distribution function (PDF) set. The procedure used for the normalization of this sample is described in reference [48]. These samples are generated with up to five light partons ($u,~d,~s$) and one $c$ quark or two heavy quarks ($c$, $b$) per event. $W+b$ production is highly suppressed and therefore negligible. A control region enriched in $W(\ell \nu )+$jets events is selected by adding a lepton requirement to the selection and is used to validate the estimate of this background. The purity of $W(\ell \nu )+$jets in the control region for SR1 (SR2) is 67 % (47 %). After full selection the contribution of $b$($c$)-quarks to the dominant $W(\ell \nu )$+jets background is approximately 39 % (38 %) for SR1 and 52 % (37 %) for SR2. The systematic uncertainty on this background is approximately 20 %. Finally, the small contribution from $t\bar{t}$ is estimated using MC samples and validated in data control regions before applying signal selection requirements. The $t\bar{t}$ process is selected with very high purity by requiring events with one lepton and large jet multiplicities.

**Fig. 4:**
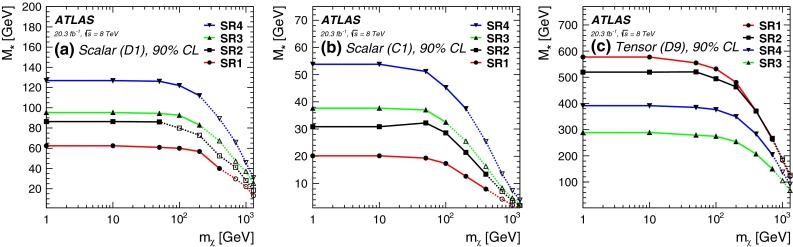
Lower limits on $M_*$ at 90 % CL for the SR1 (*red*), SR2 (*black*), SR3 (*green*), and SR4 (*blue*) as a function of $m_{\chi }$ for the operators **a** D1, **b** C1, and **c** D9. *Solid lines* and *markers* indicate the validity range of the effective field theory assuming couplings $g_q g_\chi <4\pi$, the *dashed lines* and *hollow makers* represent the full collider constraints

The dominant source of background for SR3 and SR4 is $t\bar{t}$ events. In SR3, this contribution is estimated from data using a control region not overlapping with SR4 and largely dominated by $t\bar{t}$ events with one of the two top quarks decaying semileptonically. The five-jets requirement is relaxed to three jets. Additionally, the event is required to contain exactly one lepton with $p_\mathrm{T}^{e(\mu )}>30~(25)$ GeV and must fulfill $E_\mathrm {T}^{\mathrm {miss}}+m_\mathrm {T}>25~(30)$ GeV for the electron (muon) channel. The potential signal contribution to this selection is less than 0.1 %. The uncertainties are small because the SR3 data control region uses a kinematic region similar to the signal region with the lepton veto and jet multiplicity being the main difference. These effects were studied and considered as systematic uncertainties. Dominant uncertainties are related to jets and the top quark momentum distribution. Corrections to compensate for the differences in efficiency and acceptance between hadronic and semi-leptonic top decays are derived from MC samples generated using the POWHEG BOX generator [49] interfaced with JIMMY4.31 [50] with the next-to-leading-order (NLO) PDF set CT10 [51]. The systematic uncertainty on the $t\bar{t}$ background in SR3 of approximately $7\,\%$ is derived by studying corrections for the top quark momentum distribution, and shower modelling by interfacing the same generator with PYTHIA6 [52, 53].

In SR4, the $t\bar{t}$ background is estimated from data using a control region obtained by requiring $60~\text {GeV}< m_\text {T}< 90$ GeV and loosening the selection criteria on $E_\mathrm {T}^{\mathrm {miss}}$, $am_\mathrm {T2}$, and $E_\mathrm {T}^{\mathrm {miss}}/ \sqrt{H_\text {T}^{4j}}$. A similar selection, but applying an inverted $b$-tagging requirement, is used to estimate the $W(\ell \nu )+$jets background. The uncertainty on the $t\bar{t}$ background is estimated to be approximately 20 % [24], which is larger than the uncertainty in SR3 due to the limited statistics. These uncertainties are evaluated by varying the renormalisation and factorisation scale of the simulations, comparing alternative PDF sets, and studying the effects of different shower generators and of ISR and final-state radiation.

Additional sources of background, which include single-top, $t\bar{t}+Z/W$, and diboson production, are estimated in all signal regions using simulations and NLO cross sections [54, 55]. The single-top (s-channel) and $Wt$ background is generated using the POWHEG generator. The single-top t-channel is generated with ACERMC3.8 [56] interfaced with PYTHIA6. Associated production of $t\bar{t}$ and a vector boson ($W$, $Z$) are generated with MADGRAPH5 [57] with up to two additional partons interfaced with PYTHIA6. The cross-sections for $t\bar{t}$ production in association with a $W$ ($Z$) boson are determined using the MSTW2008 NLO (CTEQ6.6M) PDF sets. The diboson samples are generated using HERWIG6.520 [58, 59] and JIMMY4.31 with the CTEQ6L1 PDF set. The multijet background is estimated using data-driven methods [60] and is found to be negligible in all signal regions after full selection.

Object reconstruction efficiencies in simulated events are corrected to reproduce the performance measured in data. The systematic uncertainty of the background estimates derived from simulation combines the uncertainties on the efficiency of the $b$-tagging algorithm, the uncertainties on the determination of the energy scale and resolution of the jet energy and $E_\mathrm {T}^{\mathrm {miss}}$, the theoretical uncertainty on the various cross-sections, changes in the shapes of distributions used to extrapolate event counts from control regions to the signal region, data driven corrections and the PDF uncertainties. Overall, the systematic uncertainty on the background estimated from simulation is calculated to be between 12 and 18 %, depending on the signal region.

The simulation of the signal samples of $pp\rightarrow \chi \overline{\chi }+ b(\overline{b})$, $pp\rightarrow \chi \overline{\chi }+ t\overline{t}$, and $b$-FDM employs the MADGRAPH5 generator interfaced with PYTHIA6 using the CTEQ6L1 PDF. Samples are generated for operators D1, C1, and D9, assuming $M_*= 1$  TeV and $m_{\chi }$ between 10 and 1300 GeV. Samples for the $b$-FDM model are generated for $m_\chi$ values between 1 and 1300 GeV and mediator masses, $m_{\phi }$, between 5 and 3000 GeV. The instrumental uncertainties on the simulated signal yields for D1, C1, and D9 operators are between 11 and 15 %, depending on the signal region. The equivalent uncertainties for the $b$-FDM model range between 6 and 16 % depending on $m_{\chi }$ and the mediator mass. The uncertainties from the PDF are computed by comparing the rates obtained with the default PDF set (CTEQ6L1) with those obtained with two alternative sets (MSTW2008LO and NNPDF21LO [61, 62]). The uncertainties on the signal acceptance from PDF and scale variations are estimated to be approximately 10 % for the D1, C1, and D9 operators for $m_{\chi }=10$ GeV and approximately $6$ % for $b$-FDM models.

The validity of the effective field theory assumption depends on the momentum transfer of the process modelled, which should be below the energy scale of the underlying interactions [63]. To account for this, the momentum transfer $m(\chi \chi ) = Q_\mathrm {tr}$ in the events is required to be less than the energy scale probed. Specifically, $Q_\mathrm {tr}$ must be smaller than the mass $M$ of the heavy mediator. For an ultraviolet completion this implies $M_*=M/\sqrt{g_q g_\chi }$. Along with perturbativity of the couplings $g_q g_\chi <4\pi$ this leads to the following validity requirements on MC truth level: $Q_\mathrm {tr}<4\pi ( M_*^3/m_q )^{1/2}$ (D1), $Q_\mathrm {tr}<4\pi M_*$ (D9), $Q_{\mathrm tr}<(4 \pi )^2 M_*^2 / m_q$ (C1).

## Results

Table 2 shows the expected background from various sources in the four signal regions as well as the observed yields in data. The expected signal yields for the operators D1, C1, and D9, as well as for the $b$-FDM model are also shown. The probabilities of the background-only hypothesis, $p$ values, for the signal regions SR1, SR2, SR3, and SR4 are 0.09, 0.29, 0.24, and 0.18, respectively. As no significant excess is observed, limits on the signal yield are set using a profile likelihood ratio test following the $CL_s$ prescription [64]. Also given is the 95 % confidence level (CL) upper limit on the number of beyond-the-SM events. The yields for the $b$-FDM model are obtained assuming $m_\chi =10$ GeV and a mediator mass $m_\phi =600$ GeV. The limit on $M_*$ for a given assumption on $m_\chi$ is determined by varying $M_*$ and scaling the number of signal events predicted by the corresponding sample generated with $M_* = 1$ TeV until it is equal to the observed upper limit on beyond-the-SM events. The corresponding production cross-section for DM produced via the D1 operator in association with $b$($t$)-quarks and $m_\chi =10$ GeV is 38 (221) fb. The cross-section for $b$-FDM models with $m_\phi =600~$ and $m_\chi =10$ GeV is 134 fb. The signal efficiency is independent of $M_{*}$.

Figure 3 shows the $E_\mathrm {T}^{\mathrm {miss}}$ distributions for (a) SR1, (b) SR2, and (d) SR4 and (c) the $R$ variable for SR3.

**Fig. 5:**
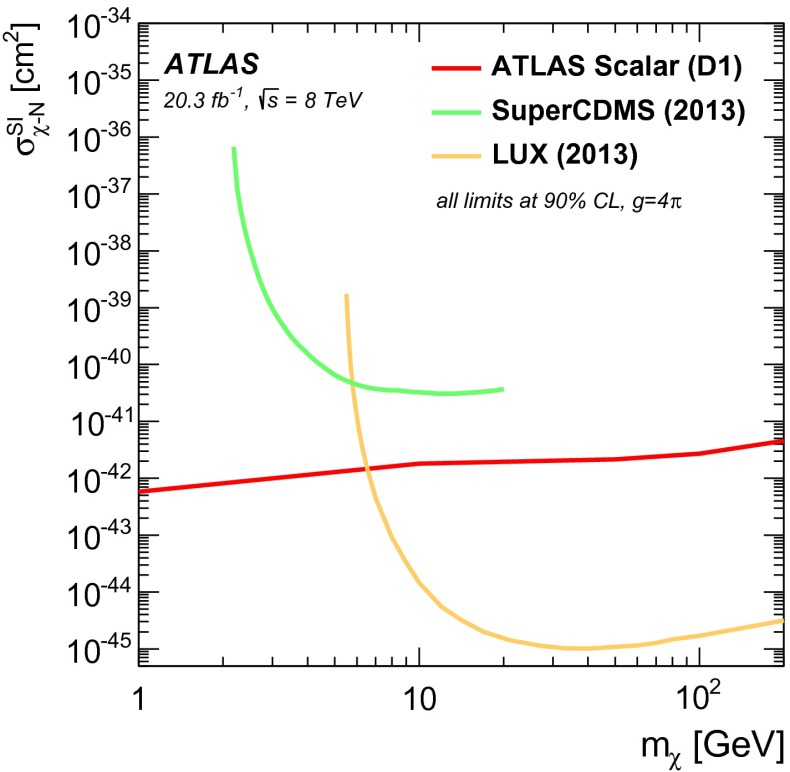
Upper limits at 90 % CL on the spin-independent $\chi$–nucleon cross-section ($\sigma _{\chi -\hbox {N}}^\mathrm{SI}$) for the scalar operator D1 (*red*) as a function of $m_{\chi }$. The *yellow and green curves* represent the exclusion limits recently set by the LUX and Super-CDMS collaborations [6, 7, 65]. The coupling is assumed to be $g_q g_\chi =g=4 \pi$

**Fig. 6:**
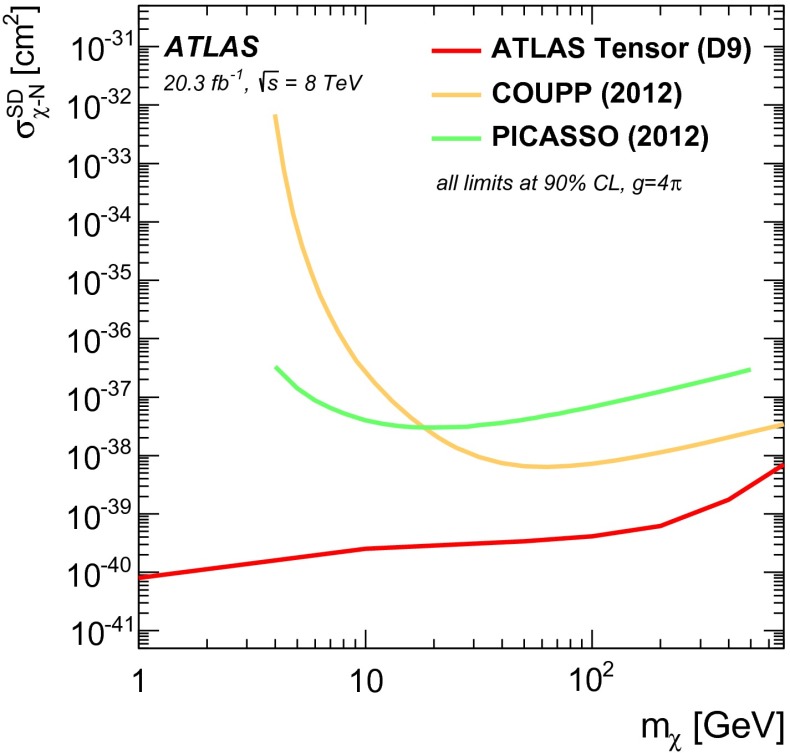
Upper limits at 90 % CL on the spin-dependent $\chi$–nucleon cross-section ($\sigma _{\chi -\hbox {N}}^\mathrm{SD}$) for the tensor operator D9 (*red*) as a function of $m_{\chi }$. The *yellow and green curves* represent the exclusion limits recently set by the COUPP and PICASSO collaborations [8, 9, 65]. The coupling is assumed to be $g_q g_\chi =g=4 \pi$

**Fig. 7:**
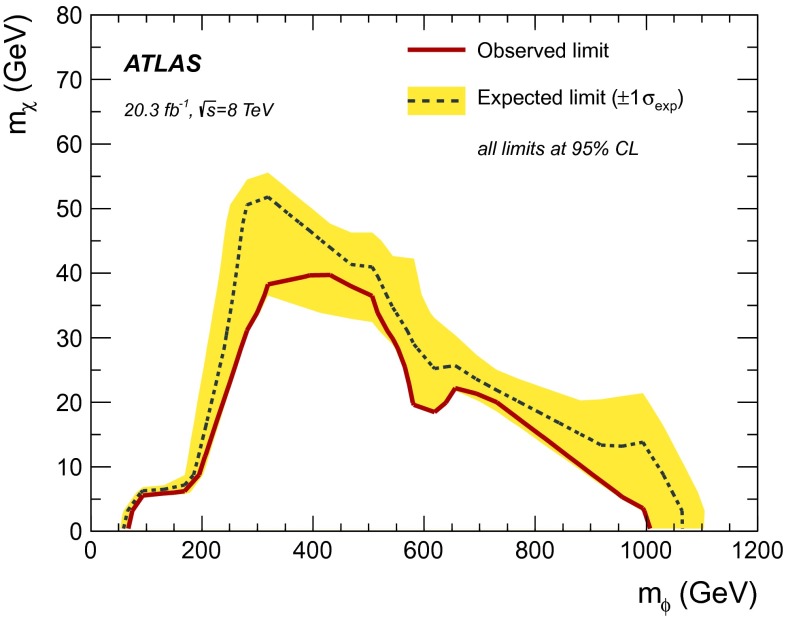
Exclusion contour at 95 % CL for the $b$-FDM model from combined results of SR1 and SR2. The expected limit is given by the *dashed line*, and the *yellow band* indicates the $\pm 1~\sigma$ uncertainty. The observed limit, largely dominated by SR1, is given by the *solid red line*. The region *beneath the curve* indicating the observed limit is excluded

Figure 4 shows the 90 % CL exclusion curves for the effective mass scale $M_*$ as a function of $m_{\chi }$. The results for the operators D1, C1, and D9 are presented individually for all four signal regions. The best limits on the D1 and C1 operators are obtained using SR4, while SR1 provides the best limits on the D9 operator, as shown in Fig. 4. These limits are then converted into limits on the $\chi$–nucleon cross-section [12]. Figures 5 and 6 show the corresponding 90 % CL exclusion curves for the spin-independent and spin-dependent $\chi$–nucleon cross-section for the scalar (D1) and tensor (D9) operators as a function of $m_{\chi }$ for the strongest results obtained in any signal region. The most stringent limits set by direct detection experiments [6–9] are also shown. Only $m_\chi$ where more then $90\,\%$ of the events fulfill the effective field theory validity constraints are shown in Figs. 5 and 6.

The limits shown are especially strong in the low-mass region where several collaborations [28, 66–68] have recently claimed possible observations of DM. The results reported in this article represent the first ATLAS limits on the scalar operator C1 and they significantly improve the sensitivity to $\chi$–nucleon interactions mediated by the scalar operator D1 compared to previous ATLAS results [14, 16, 18, 19].

Figure 7 shows the exclusion curves observed and expected for the $b$-FDM model as a function of the mediator and DM masses. For each point in ($m_\chi$, $m_\phi$), the signal region with the best expected sensitivity is used, with SR1 dominating over the other signal regions. For a DM particle of approximately 35 GeV, as suggested by the interpretation of data recorded by the Fermi-LAT collaboration, mediator masses between approximately 300 and 500 GeV are excluded at 95 % CL.

## Conclusions

In summary, this article reports a search for dark-matter pair production in association with bottom or top quarks. The analysis is performed using $20.3 \mathrm {~fb}^{-1}$ of $pp$ collisions collected at $\sqrt{s} = 8$ TeV by the ATLAS detector at the LHC. The results are interpreted in the framework of an effective field theory to set stringent limits on scalar and tensor interactions between Standard Model and DM particles. The data are found to be consistent with the Standard Model expectations, and limits are set on the mass scale of effective field theories that describe scalar and tensor interactions between DM and Standard Model particles. The exclusion limits are strongest at low DM masses. The limit on the $\chi$–nucleon cross-section mediated by the D1 operator is improved significantly with respect to previously published ATLAS results by obtaining sensitivities of approximately $\sigma _{\chi -\hbox {N}}^\mathrm{SI}=10^{-42}\,\text {~cm}^2$ for $m_\chi =10$ GeV. Constraints on $b$-Flavoured Dark Matter models, suitable to explain a possible signal of annihilating DM, are also presented. The excluded regions depend on $m_\chi$ and $m_\phi$. For $m_\chi =35$ GeV, mediator particles with $m_\phi =300$–$500$ GeV are excluded.

## Footnotes

1 ATLAS uses a right-handed coordinate system with its origin at the nominal interaction point (IP) in the centre of the detector, and the $z$-axis along the beam line. The $x$-axis points from the IP to the centre of the LHC ring, and the $y$-axis points upwards. Cylindrical coordinates ($r,\ \phi$) are used in the transverse plane, $\phi$ being the azimuthal angle around the beam line. The pseudorapidity $\eta$ is defined in terms of the polar angle $\theta$ as $\eta =-\ln \tan (\theta /2)$. Observables labeled “transverse” are projected into the $x$–$y$ plane.

2 Since the longitudinal component of the momentum of the neutrinos is not measured, the measured properties of the $W$ boson candidates are limited to their transverse momentum and transverse mass, defined as $m_\mathrm {T}=\sqrt{ (E_\mathrm {T}^{\mathrm {miss}}+ p_\mathrm {T}^\ell )^2 - (E^{\mathrm {miss}}_x\! + p_x^\ell )^2 - (E^{\mathrm {miss}}_y\! + p_y^\ell )^2}$ where $E_\mathrm {T}^{\mathrm {miss}}$ is the magnitude of the missing transverse momentum vector, $p_\mathrm {T}^\ell$ is the transverse momentum of the lepton and $p_x^\ell$ and $p_y^\ell$ ($E^{\mathrm {miss}}_x\!$ and $E^{\mathrm {miss}}_y\!$) are the magnitude of the $x$ and $y$ components of the lepton momentum (missing transverse momentum) respectively.
